# *BnVTE2*-Mediated Vitamin E Modulation Is Associated with Antioxidant Capacity, Fatty Acid Stability, and Seed Longevity in *Brassica napus*

**DOI:** 10.3390/plants15182757

**Published:** 2026-09-09

**Authors:** Qiao Ruan, Xiaoli Tan, Wajahat Hussain, Yunxia Zeng, Yonghong Zhou

**Affiliations:** 1 Chongqing Chemical Industry Vocational College, Putidong Road 2009, Changshou District, Chongqing 401228, China; 2 College of Agronomy and Biotechnology, Southwest University, Tiansheng Road 2, Beibei District, Chongqing 400715, China; wajahat7253@yahoo.com (W.H.);

**Keywords:** vitamin E, tocopherol, *VTE2*, fatty acids, *Brassica napus*, seed longevity, antioxidant capacity

## Abstract

Vitamin E (tocopherols) is an important lipid-soluble antioxidant that contributes to plant redox homeostasis and seed longevity. To investigate relationships among *BnVTE2*-dependent tocopherol accumulation, antioxidant capacity, fatty acid stability, and seed storability in rapeseed, we generated *Brassica napus* lines overexpressing *BnVTE2* (OE) and lines with RNA interference-mediated suppression of *BnVTE2* (RNAi). *BnVTE2* encodes homogentisate phytyltransferase, a key enzyme catalyzing the committed step in tocopherol biosynthesis. Modulation of *BnVTE2* expression resulted in corresponding changes in total tocopherol content. OE lines exhibited higher superoxide dismutase (SOD), peroxidase (POD), and catalase (CAT) activities, lower H_2_O_2_ and malondialdehyde (MDA) accumulation, and improved seed performance after long-term storage, whereas RNAi lines showed the opposite trends. Transcriptome and network analyses identified lipid-related transcriptional changes and glycerolipid-associated candidate hubs; enrichments in phenylpropanoid biosynthesis and hormone signaling were treated as broader secondary responses rather than direct components of tocopherol metabolism. After 42 months of storage, seeds with higher tocopherol levels retained greater germination capacity and showed reduced lipid peroxidation relative to tocopherol-deficient lines. Together, these findings support an association among *BnVTE2*-mediated tocopherol accumulation, antioxidant defense, fatty acid stability, and seed longevity in *B. napus*. Direct validation of glycerolipid remodeling and hub-gene function will be required in future studies.

## 1. Introduction

Vitamin E comprises lipid-soluble tocochromanols that function as antioxidants in plants and are important dietary nutrients. In oilseed crops, tocopherols are abundant in mature seeds, where their composition varies with genotype, development, and environment. In *Brassica napus*, γ- and α-tocopherols are the major seed forms, and recent genetic and developmental studies have confirmed substantial variation in vitamin E accumulation among rapeseed genotypes and seed developmental stages [1,2].

Core tocopherol biosynthetic genes were originally defined in Arabidopsis thaliana and subsequently characterized in oil crops. *VTE2* encodes homogentisate phytyltransferase, which catalyzes condensation of homogentisic acid (HGA) with phytyl diphosphate to form 2-methyl-6-phytylbenzoquinone (MPBQ), a committed step influencing total tocopherol accumulation [3]. *VTE1*, *VTE3*, and *VTE4* subsequently control cyclization and methylation steps that determine tocopherol composition [4,5]. Recent studies have extended this framework by demonstrating contributions from chlorophyll-derived phytol recycling and associated kinases to tocopherol biosynthesis, and by identifying natural variation in BnaC02.VTE4 that alters vitamin E accumulation in rapeseed [1,2,6,7].

Tocopherol biosynthesis and triacylglycerol (TAG) synthesis are distinct metabolic processes, but tocopherols can protect unsaturated membrane and storage lipids from non-enzymatic peroxidation. This antioxidant function provides a plausible basis for associations between tocopherol status and fatty-acid stability during seed maturation, storage, and germination. In *B. napus*, genetic modification of tocopherol composition has also been associated with changes in fatty-acid accumulation during seed germination [8].

Earlier crop studies reported genotype- and environment-dependent relationships between tocopherol and fatty-acid profiles [9], whereas recent *B. napus* manipulation of tocopherol composition likewise altered fatty-acid accumulation during germination [8]. These observations indicate that simple correlations can be confounded by genetic and environmental effects. Therefore, transgenic materials with contrasting endogenous tocopherol levels provide a useful approach for testing whether tocopherol status is consistently associated with fatty-acid composition and oxidative stability in the same genetic background.

During dry storage, tocopherols are particularly important because they can interrupt lipid-peroxidation chain reactions in lipid-rich seeds. Foundational Arabidopsis *vte2* studies showed reduced seed longevity and increased oxidized lipids in tocopherol-deficient seeds [10]. More recent work on seed longevity has likewise emphasized the importance of lipid droplet homeostasis and protection of stored cellular components during seed aging [11].

Vitamin E is therefore relevant to both seed antioxidant protection and maintenance of lipid quality, but the relationships among *BnVTE2*-dependent tocopherol accumulation, redox status, fatty-acid stability, and long-term seed viability remain incompletely resolved in polyploid *B. napus*. In this study, vitamin E levels were modulated by *BnVTE2* overexpression (OE) or RNA interference (RNAi). We combined physiological and biochemical measurements with transcriptome and network analyses to determine (i) how *BnVTE2* expression relates to tocopherol accumulation, antioxidant enzyme activity, and ROS levels; (ii) whether altered tocopherol status is associated with fatty-acid composition and lipid-related transcriptional changes; and (iii) whether higher tocopherol accumulation is associated with improved germination and reduced lipid peroxidation after long-term storage. Broader transcriptomic enrichments outside lipid/redox pathways were considered secondary responses and were not interpreted as direct *BnVTE2*-controlled metabolic mechanisms.

## 2. Results

### 2.1. BnVTE2 Expression Level Is Associated with Tocopherol Content and Antioxidant Activity in Seeds

*BnVTE2* encodes homogentisate phytyltransferase, a key enzyme in tocopherol biosynthesis. To examine the relationship between tocopherol level and antioxidant responses during seed development, the full-length *BnVTE2* coding sequence was introduced into rapeseed (*Brassica napus* cv. Westar) to generate OE lines. A *BnVTE2*-specific fragment (nucleotides 605–850 of the coding sequence) was used to generate RNAi lines. Third-generation (T3) homozygous OE and RNAi plants were selected for subsequent analyses.

RT-qPCR analysis confirmed the expected increase in *BnVTE2* expression in OE lines and reduction in RNAi lines (Figure S1a,b). Lines OE-3, OE-24, OE-29, OE-37 and RNAi-8, RNAi-15, RNAi-30, and RNAi-35 were selected for subsequent biochemical analyses. Total tocopherol content was higher in OE lines and lower in RNAi lines than in WT across the examined developmental stages (Figure S1c).

**Figure 1:**
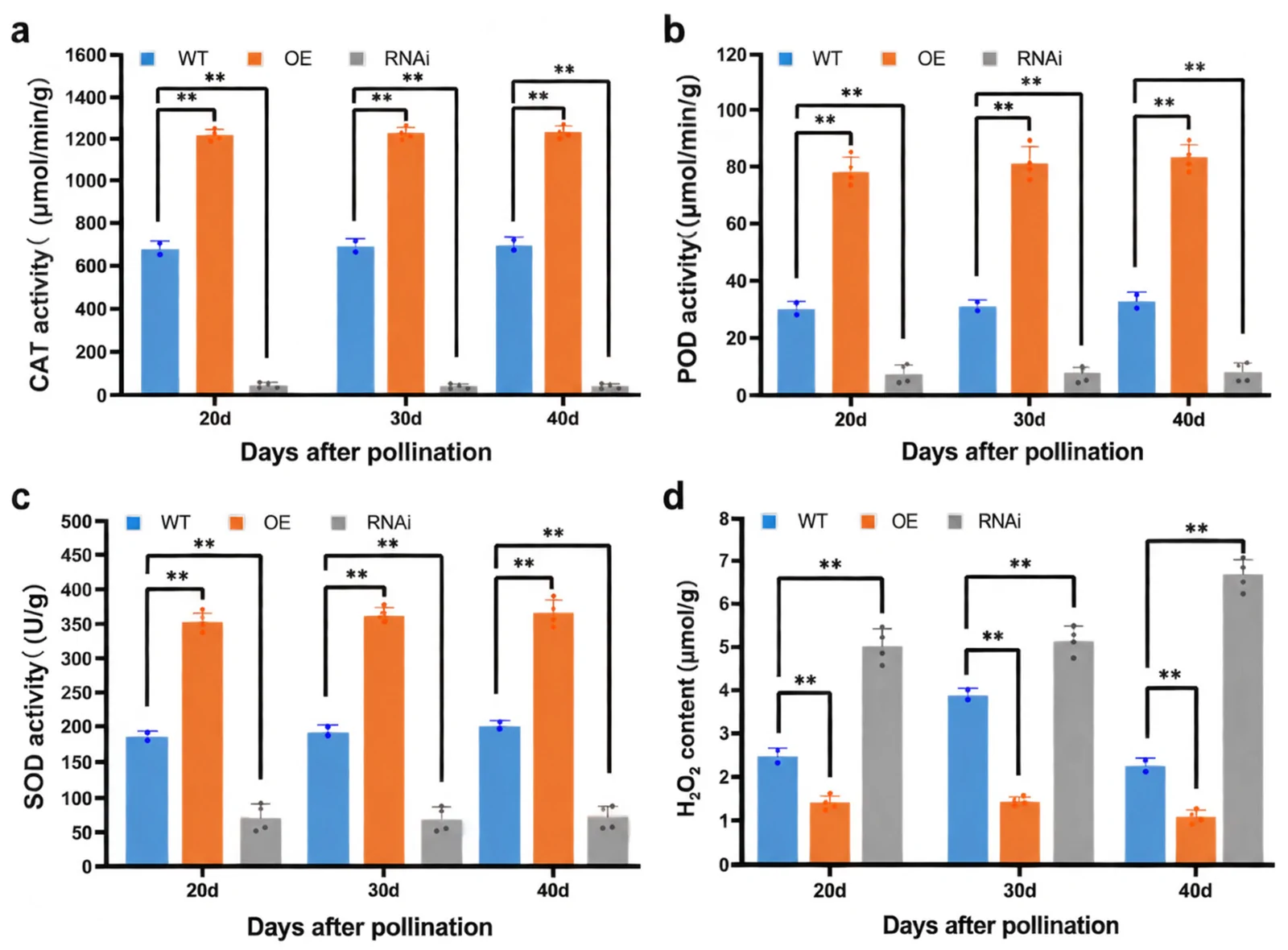
Antioxidant enzyme activities and H_2_O_2_ accumulation in *BnVTE2* transgenic seeds. (**a**–**c**) Activities of CAT (**a**), POD (**b**), and SOD (**c**) in seeds at 20, 30, and 40 days after pollination. (**d**) H_2_O_2_ content at the same developmental stages. Asterisks indicate significant differences as defined in the statistical analysis (** *p* < 0.01); Individual dots represent biological replicates.

The activities of catalase (CAT), peroxidase (POD), and superoxide dismutase (SOD) were measured in developing seeds at 20, 30, and 40 days after pollination (DAP) (Figure 1a–c). At all three developmental stages, OE seeds showed higher CAT, POD, and SOD activities than WT, whereas RNAi seeds showed lower activities. In contrast, H_2_O_2_ accumulation showed the opposite pattern, with lower H_2_O_2_ levels in OE seeds and higher levels in RNAi seeds relative to WT (Figure 1d). These results indicate that differences in *BnVTE2*-dependent tocopherol accumulation were associated with corresponding differences in antioxidant enzyme activities and H_2_O_2_ accumulation.

### 2.2. Fatty Acid Stability, Seed Germination, and Lipid Peroxidation During Long-Term Storage

**Figure 2:**
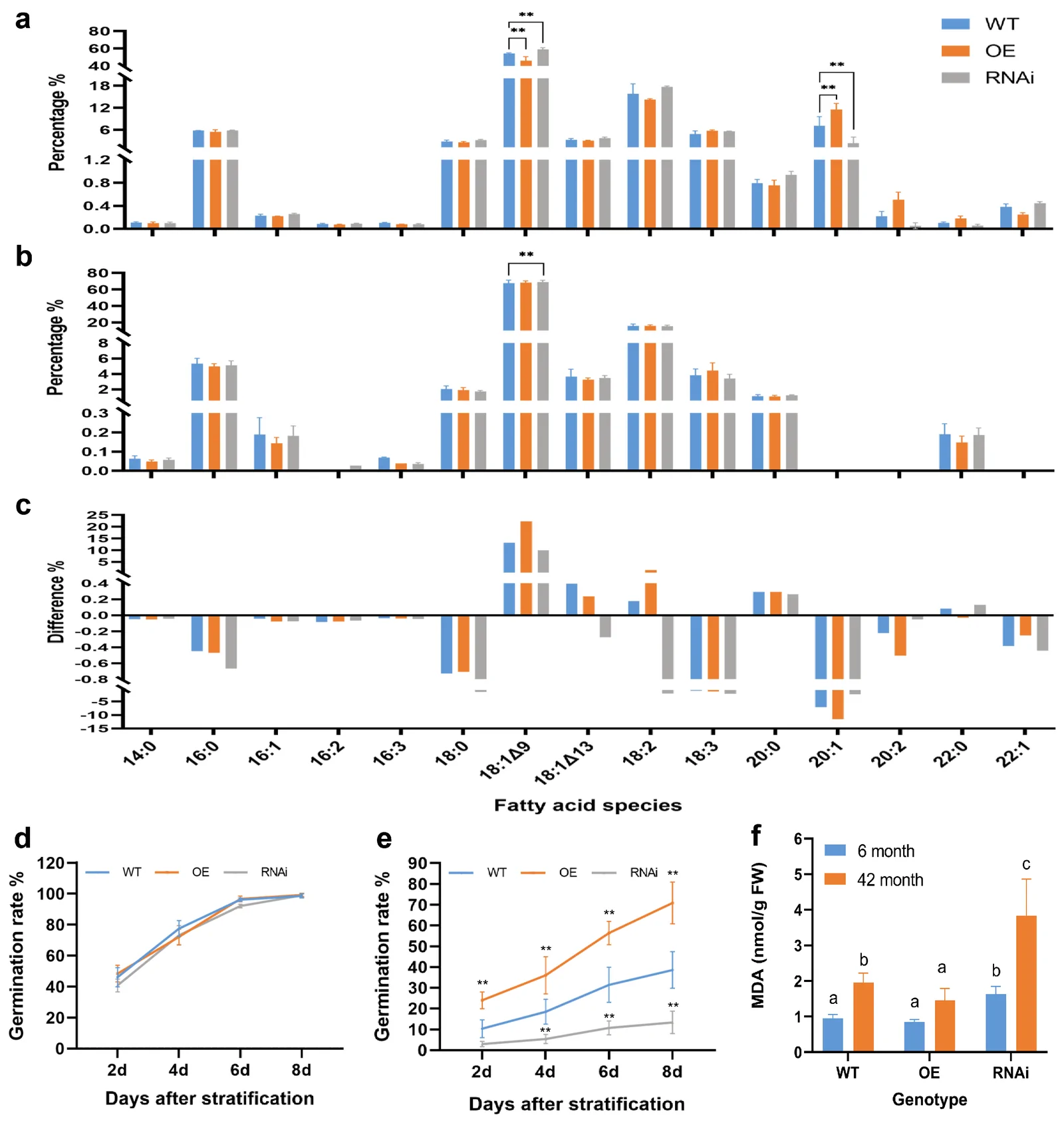
Fatty acid composition, germination rate, and MDA content in seeds and germinated seedlings. (**a**,**b**) Relative fatty-acid composition in seeds stored for 6 months (**a**) and 42 months (**b**). (**c**) Change in relative fatty-acid composition calculated as the 42-month percentage minus the corresponding 6-month percentage (percentage-point difference); negative values indicate lower relative abundance after prolonged storage. (**d**,**e**) Germination rates of seeds stored for 6 months (**d**) and 42 months (**e**). (** *p* < 0.01) (**f**) MDA content in seedlings 2 days after stratification. Different lowercase letters above the MDA bars indicate significant differences among genotypes within the same storage duration.

To compare fatty-acid composition among genotypes with contrasting tocopherol levels, the relative fatty-acid composition of seeds stored for 6 and 42 months was analyzed. As shown in Figure 2a, fatty acids with 14–22 carbons were detected in seeds stored for 6 months. The major fatty acids (>1% of total detected fatty acids) were C16:0, C18:0, C18:1, C18:2, C18:3, C20:1, and C22:1. Oleic acid (C18:1) was the most abundant fatty acid in all samples, followed by linoleic acid (C18:2). Relative to WT, OE lines showed lower proportions of C18:1 and C18:2 and higher proportions of C18:3, C20:1, and C20:2, whereas RNAi lines generally showed profiles closer to WT.

To assess fatty-acid composition after prolonged storage, seeds stored for 42 months were analyzed (Figure 2b). The overall fatty-acid profile differed from that observed after 6 months of storage, but no obvious differences were observed among WT, OE, and RNAi genotypes at 42 months. C18 fatty acids remained dominant. Minor fatty acids, including C16:2, C20:1, C20:2, and C22:1, were not detected in any of the three genotypes.

Figure 2c shows the change in relative fatty-acid composition calculated as the 42-month value minus the corresponding 6-month value. Most fatty acids showed negative changes, whereas oleic acid (C18:1) increased in all three genotypes. Linolenic acid (C18:3) and eicosenoic acid (C20:1) showed the largest declines. The decreases in the polyunsaturated fatty acids C18:2 and C18:3 were greatest in RNAi lines, consistent with greater oxidative loss in tocopherol-deficient seeds. Because these data are relative percentages rather than absolute fatty-acid concentrations, the differences should be interpreted as compositional shifts rather than direct measurements of fatty-acid synthesis or degradation.

Germination was evaluated for seeds stored for 6 and 42 months. Germination after 6 months was similar among WT, OE, and RNAi lines, with almost 100% germination by 8 days after stratification (Figure 2d). After 42 months of storage, germination rates were 39%, 71%, and 13% for WT, OE, and RNAi lines, respectively (Figure 2e). Higher vitamin E levels were associated with improved germination after prolonged storage. This pattern supports a protective role of tocopherols during prolonged storage, although the natural-aging comparison alone does not establish direct causality.

Malondialdehyde (MDA) content was measured in seedlings 2 days after stratification to evaluate lipid peroxidation after storage (Figure 2f). Direct genotype comparisons were performed separately at each storage duration using Welch’s one-way ANOVA followed by Games-Howell pairwise tests because the group variances were unequal. At 6 months, MDA differed among genotypes (*p* < 0.001); RNAi seedlings had higher MDA than WT (*p* < 0.001) and OE (*p* < 0.001), whereas WT and OE did not differ significantly (*p* = 0.194). At 42 months, MDA again differed among genotypes (*p* = 0.0015); RNAi seedlings had higher MDA than WT (*p* = 0.013) and OE (*p* = 0.004), and WT was modestly higher than OE (*p* = 0.045). These comparisons support greater lipid peroxidation in the RNAi material after both storage periods, with the strongest MDA accumulation after long-term storage.

Taken together, the storage phenotypes provide the physiological basis for the subsequent transcriptomic and network analyses, which were used to identify molecular processes associated with the observed antioxidant and lipid-stability differences.

### 2.3. Transcriptome Profiling Reveals Global Expression Dynamics and Line-Specific Patterns in BnVTE2 Transgenic Brassica napus

To investigate the global transcriptional responses associated with *BnVTE2* manipulation, RNA sequencing (RNA-seq) was performed using 30-day-after-pollination (DAP) seed samples from OE, RNAi, and wild-type (WT) lines. Clean reads were aligned to the *B. napus* reference genome with HISAT2 (v2.0.1-beta), and mapping statistics are summarized in Table S3. More than 88% of mapped reads aligned to coding sequences (CDS), while 2.0–2.3% and 7.9–9.1% corresponded to intronic and intergenic regions, respectively, a pattern typical of poly(A)-enriched libraries (Figure S2a). Mapping rates ranged from 90.72% (Ri1) to 92.43% (OE2), averaging 91.64%, with most reads uniquely mapped and paired-end, confirming high alignment quality. Boxplot analysis of log_2_(FPKM) expression distributions demonstrated excellent reproducibility among biological replicates (Figure S2b). Median log_2_(FPKM) values were consistent across all groups, with slightly higher medians observed in RNAi lines (Ri1–Ri3). Low intra-group variability indicated reliable expression quantification.

**Figure 3:**
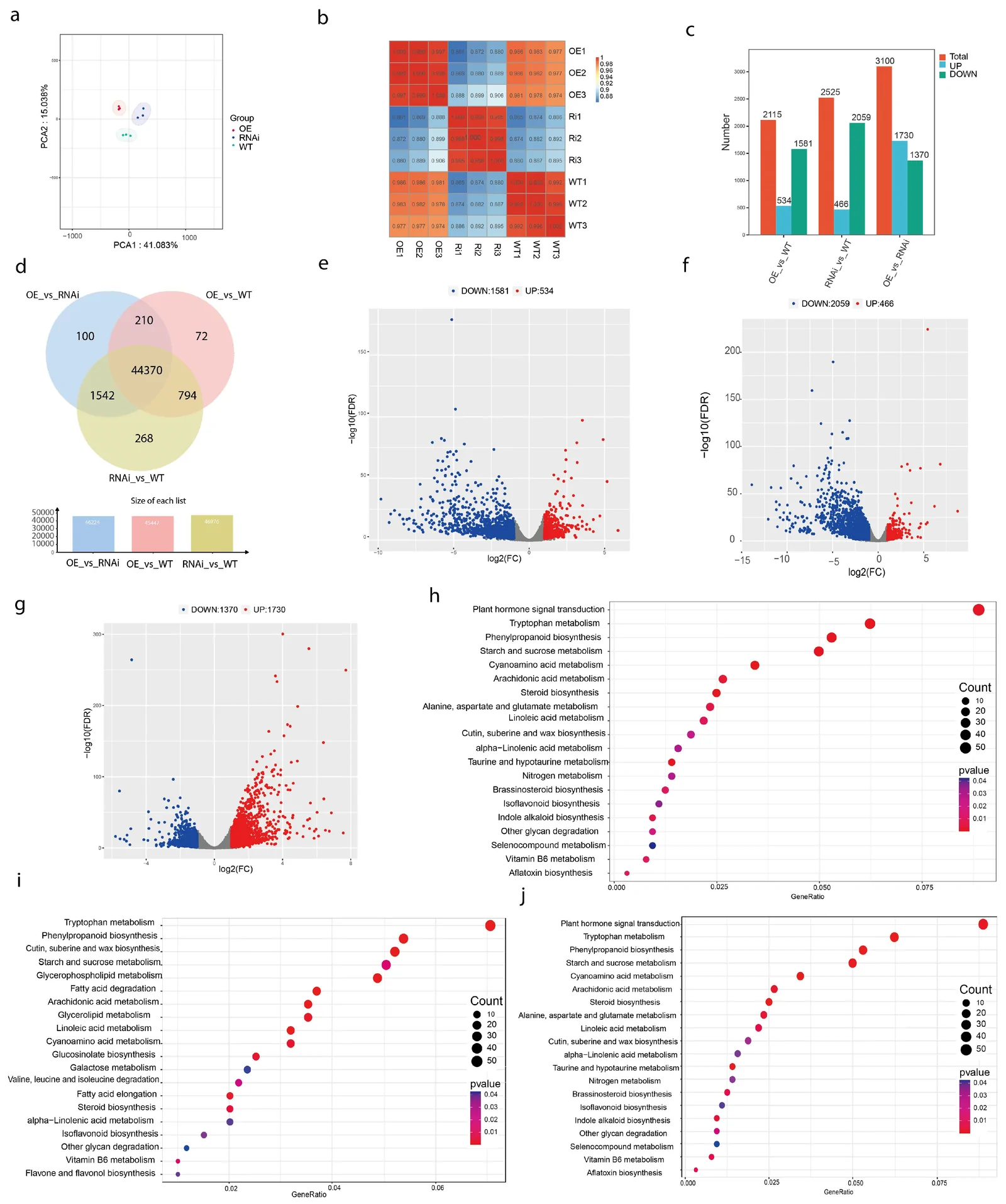
Global transcriptome profiling and functional enrichment in *BnVTE2* transgenic lines. (**a**) Principal component analysis (PCA) of biological replicates from OE, RNAi, and WT lines based on transcriptome-wide gene expression profiles. (**b**) Pearson correlation analysis showing high within-group reproducibility and separation among genotypic groups. (**c**) Numbers of upregulated (red) and downregulated (blue) differentially expressed genes (DEGs) identified in each pairwise comparison. (**d**) Venn diagram showing the overlap among DEGs identified in OE vs. WT, RNAi vs. WT, and OE vs. RNAi using the same differential-expression criteria applied in the DEG analysis. (**e**–**g**) Volcano plots showing significantly upregulated genes in red, significantly downregulated genes in blue, and genes not meeting the differential-expression significance criteria in gray (|log_2_FC| > 1, FDR ≤ 0.05) for (**e**) OE vs. WT, (**f**) RNAi vs. WT, and (**g**) OE vs. RNAi. (**h**–**j**) KEGG pathway enrichment analysis of DEGs in (**h**) OE vs. WT, (**i**) RNAi vs. WT, and (**j**) OE vs. RNAi.

Principal component analysis (PCA) revealed distinct clustering of OE, RNAi, and WT samples (Figure 3a), while Pearson correlation analysis (Figure 3b) showed strong intra-group correlations and lower inter-group correlations, indicating genotype-specific transcriptomic divergence. Differential expression analysis identified 2115 differentially expressed genes (DEGs) in OE vs. WT, 2525 DEGs in RNAi vs. WT, and 3100 DEGs in OE vs. RNAi (Figure 3c). Volcano plots further illustrated the distribution of significantly regulated genes in each comparison (Figure 3e–g). In OE vs. WT, 534 genes were significantly upregulated and 1581 were downregulated. In RNAi vs. WT, 466 genes were upregulated and 2059 were downregulated. The OE vs. RNAi comparison showed 1730 upregulated and 1370 downregulated genes. To examine the relationship among transcriptional responses associated with *BnVTE2* OE and RNAi, DEG lists from the three pairwise comparisons were compared using the same differential-expression threshold. The Venn diagram was regenerated to show the corrected overlap among OE vs. WT, RNAi vs. WT, and OE vs. RNAi comparisons (Figure 3d). Overall, these results indicate that modulation of *BnVTE2* expression caused substantial transcriptomic reprogramming, with both shared and genotype-specific responses during seed development.

### 2.4. Functional Enrichment Analysis of Differentially Expressed Genes

To characterize the biological functions and metabolic pathways associated with *BnVTE2*-dependent transcriptional changes, GO and KEGG enrichment analyses were conducted separately for the DEGs identified in the OE vs. WT, RNAi vs. WT, and OE vs. RNAi comparisons (Figure S2c–e and Figure 3h–j).

GO analysis showed that OE vs. WT DEGs were enriched in response to water, response to water deprivation, phenylpropanoid metabolism/biosynthesis, suberin biosynthesis, plant-type cell wall, glyoxysome, oxidoreductase activity, and lipid-binding terms (Figure S2c). RNAi vs. WT DEGs were enriched in seed maturation, lipid storage, suberin biosynthesis, response to water deprivation, lipid droplet, aleurone grain, storage vacuole, nutrient reservoir activity, and monooxygenase activity (Figure S2d). OE vs. RNAi DEGs were enriched in seed maturation, lipid storage, defense response to insects, amino-acid import, plasma membrane, cell wall, microtubule-associated complexes, microtubule motor activity, and ATP-dependent transport terms (Figure S2e).

KEGG enrichment identified lipid-related, carbohydrate, amino-acid, secondary-metabolism, and signaling pathways in all three comparisons (Figure 3h–j).

In OE vs. WT, enriched pathways included glycerolipid and glycerophospholipid metabolism, linoleic and α-linolenic acid metabolism, cutin/suberin/wax biosynthesis, starch and sucrose metabolism, plant hormone signal transduction, phenylpropanoid biosynthesis, tryptophan metabolism, glucosinolate/flavonoid/isoflavonoid biosynthesis, ABC transporters, and vitamin B6 metabolism (Figure 3h). In RNAi vs. WT, enriched pathways included glycerolipid/glycerophospholipid metabolism, fatty-acid degradation/elongation, linoleic and α-linolenic acid metabolism, starch and sucrose metabolism, cutin/suberin/wax biosynthesis, tryptophan metabolism, phenylpropanoid biosynthesis, amino-acid degradation, steroid biosynthesis, and vitamin B6 metabolism (Figure 3i). In OE vs. RNAi, enriched pathways included fatty-acid-related pathways, starch and sucrose metabolism, cutin/suberin/wax biosynthesis, plant hormone signal transduction, tryptophan metabolism, phenylpropanoid biosynthesis, amino-acid metabolism, nitrogen metabolism, steroid/brassinosteroid biosynthesis, isoflavonoid biosynthesis, and indole alkaloid biosynthesis (Figure 3j).

These enrichment results provide a descriptive overview of the transcriptomic response. Lipid-related pathways were examined further because they directly matched the biochemical focus of this study; phenylpropanoid and hormone-signaling enrichments are reported as secondary transcriptomic outcomes.

### 2.5. Lipid Metabolic Pathways and Associated Transcriptomic Responses During Seed Development

Based on the KEGG enrichment results, five lipid-related pathways—α-linolenic acid metabolism, linoleic acid metabolism, glycerophospholipid metabolism, glycerolipid metabolism, and fatty-acid degradation—were selected for detailed analysis. Phenylpropanoid biosynthesis was included as an additional significantly enriched pathway. Representative KEGG pathway maps are provided in the Supplementary Materials (Figures S3–S8).

Among the selected genes in these pathways (Table S2), phenylpropanoid biosynthesis included strongly downregulated peroxidase genes in OE lines, including BnaC02g10690D (log_2_FC = −5.264), together with differentially expressed HCT- and COMT-annotated genes. In α-linolenic- and linoleic-acid metabolism, OPCL1-, AOC-, and DOX-annotated genes showed genotype-dependent differential expression, whereas selected ADH1- and LOX1-annotated genes were downregulated. In glycerophospholipid metabolism, several PHOSPHO1-annotated genes showed differential expression; notably, BnaC03g78050D, encoding lysophospholipase II, was downregulated in OE but upregulated in RNAi relative to WT. In glycerolipid metabolism, BnaA02g33390D (AKR1) was upregulated in OE, whereas glycerol-kinase genes, including BnaA02g19720D and BnaA07g35620D, were downregulated. In the fatty-acid degradation pathway, most of the selected ADH1-, ALDH-, and ACSL-annotated genes were downregulated, whereas BnaA04g01830D, encoding citrate synthase, was upregulated in OE and BnaA05g31340D (ACSL) was upregulated in RNAi.s.

Thus, the detailed pathway analysis documented genotype-dependent expression differences in multiple lipid-related pathways, together with a separate phenylpropanoid response.

### 2.6. Hub Gene Network Identifies Glycerolipid-Related Genes as Candidate Network Hubs

**Figure 4:**
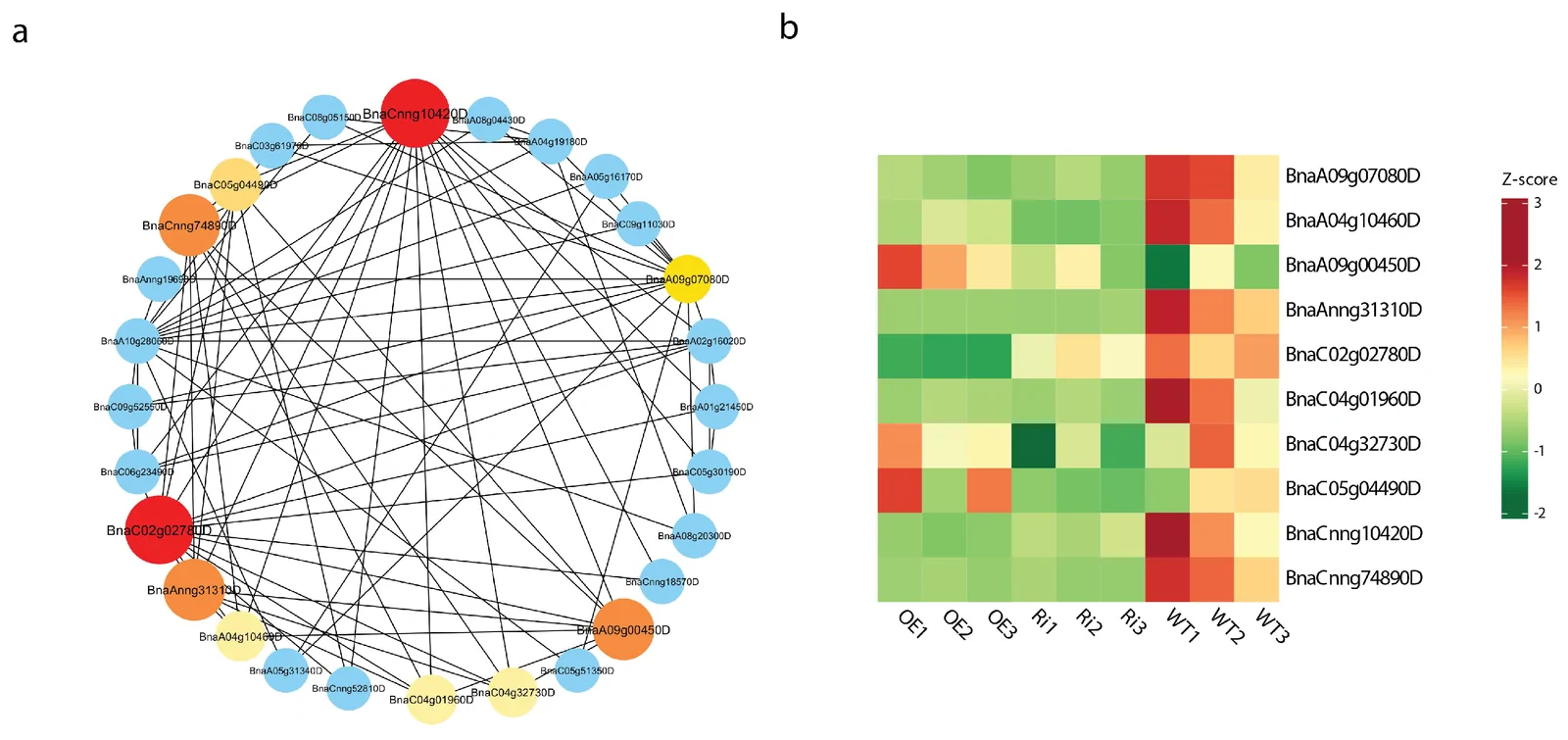
Hub Gene Network and Expression Analysis. (**a**) PPI network of DEGs across six selected pathways, constructed using STRING (confidence score > 0.4) and visualized in Cytoscape v3.10. Node color denotes cytoHubba/MCC ranking: red marks the highest-ranked hub nodes, orange and yellow shades denote the remaining top-ranked hub nodes, and blue denotes the other network nodes; node size increases with network connectivity. (**b**) Heatmap of the top 10 hub genes identified by cytoHubba (MCC ranking).

To explore interactions among all DEGs across the six selected pathways, a protein-protein interaction (PPI) network was constructed using STRING (confidence score > 0.4) and visualized in Cytoscape v3.10. The integrated network showed extensive connectivity among genes involved in glycerolipid metabolism, fatty-acid degradation, and phenylpropanoid biosynthesis (Figure 4a). This network connectivity identifies candidate associations among these pathways but does not demonstrate that *BnVTE2* or tocopherol directly regulates lipid metabolism.

Hub gene analysis using cytoHubba (MCC ranking) identified the top 10 hub genes (Table S4). Nine of the top ten hubs (90%) were annotated to glycerolipid metabolism, including TGL4 (*BnaC02g02780D*, *BnaCnng10420D*), *GPAT* (*BnaA09g00450D*, *BnaAnng31310D*, *BnaCnng74890D*, *BnaC05g04490D*), and *GPD1* (*BnaA04g10460D*, *BnaC04g01960D*, *BnaC04g32730D*). Most of these genes were downregulated in OE and RNAi lines (Figure 4b), indicating altered expression of glycerolipid-related genes rather than direct evidence of altered TAG flux. The remaining hub, *ACOX1 (BnaA09g07080D)*, is annotated to fatty-acid β-oxidation and links the network to fatty-acid catabolism.

Collectively, these in silico analyses identify glycerolipid metabolism as a candidate network-associated pathway linked to *BnVTE2*-mediated changes. Because TAG content, membrane lipid composition, and lipid flux were not measured directly, these associations should not be interpreted as evidence that *BnVTE2* directly regulates lipid metabolism.

### 2.7. Analysis of Transcription Factors (TFs) Associated with BnVTE2 Modulation

**Figure 5:**
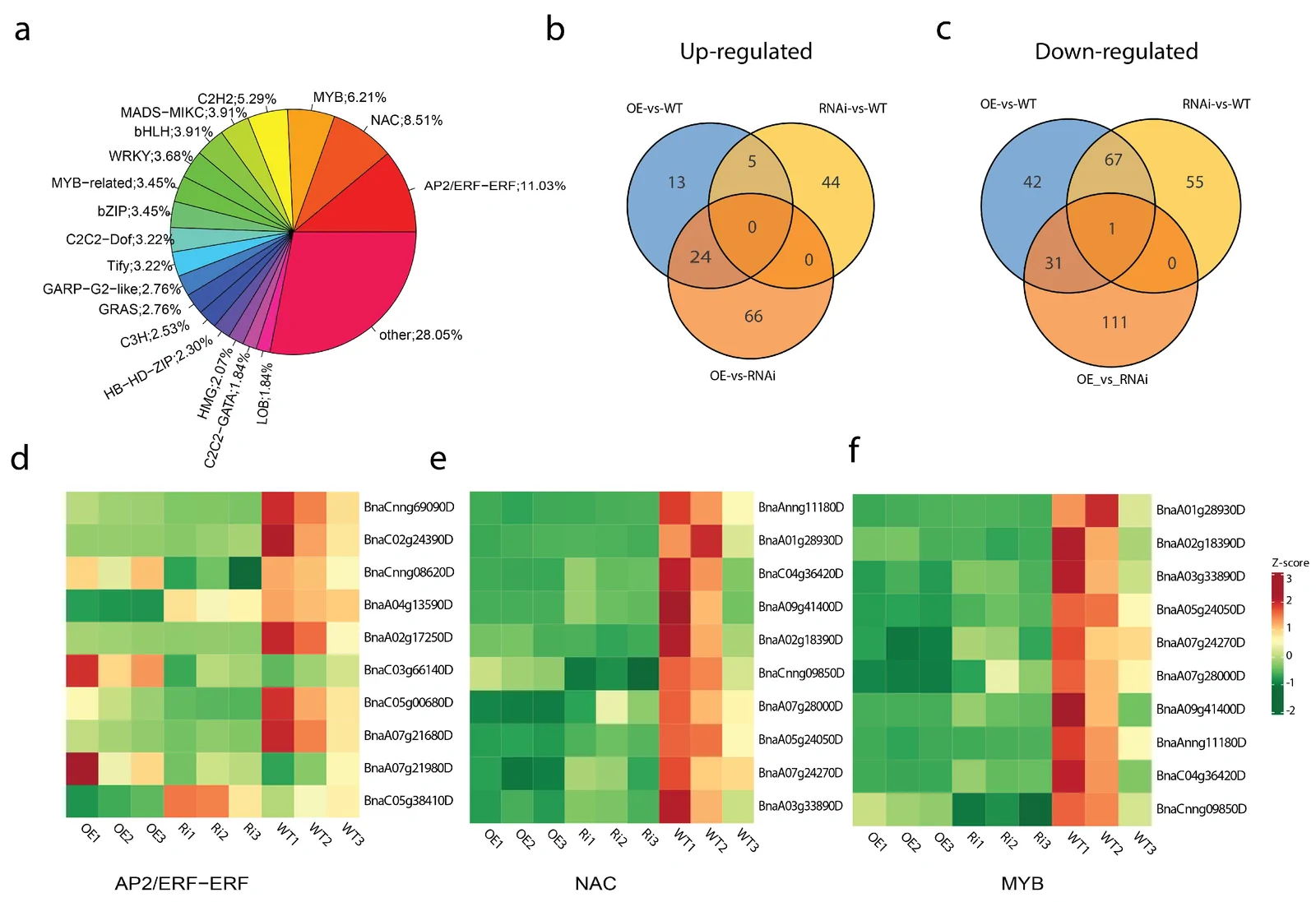
Transcription Factor (TF) Analysis in Response to *BnVTE2* Modulation. (**a**) Pie chart showing the distribution of differentially expressed TFs. (**b**) Venn diagram of upregulated TFs. (**c**) Venn diagram of downregulated TFs. (**d**–**f**) Heatmaps of the top ten differentially expressed *AP2/ERF-ERF*, *NAC*, and *MYB* family members, respectively.

A total of 318 transcription factors (TFs) were differentially expressed among the three genotypes, as determined from the transcriptomic analysis. The TF family distribution (Figure 5a) showed *AP2/ERF-ERF* as the most abundant family with 48 TFs (11.03%), followed by *NAC* (37; 8.51%), *MYB* (27; 6.21%), and *C2H2* (23; 5.29%). Other families, including *MADS-MIKC*, *bHLH*, *WRKY*, *bZIP*, and others, were less represented, while minor families collectively categorized as others accounted for 28.05% of the total.

Among the up-regulated TFs, 13, 44, and 66 were identified in OE-vs-WT, RNAi-vs-WT, and OE-vs-RNAi comparisons, respectively, with 5 shared between OE-vs-WT and RNAi-vs-WT, and 24 between OE-vs-WT and OE-vs-RNAi (Figure 5b). For the down-regulated TFs, 42, 67, and 111 were identified in OE-vs-WT, RNAi-vs-WT, and OE-vs-RNAi, respectively, with 31 shared between OE-vs-WT and RNAi-vs-WT, and 1 between OE-vs-WT and OE-vs-RNAi (Figure 5c). These overlaps suggest that modulating *BnVTE2* expression triggers both common and distinct transcriptional responses depending on the tocopherol levels.

Heatmap visualization showed contrasting expression patterns within three major TF families (Figure 5d–f). AP2/ERF-ERF family members were generally expressed at higher levels in OE lines and at lower levels in RNAi lines. Several NAC TFs also differed between OE and RNAi plants, and many MYB TFs showed higher expression in OE than in RNAi lines. These patterns identify TF families associated with the *BnVTE2*/tocopherol phenotype but do not establish direct regulation by tocopherols.

### 2.8. WGCNA Trait-Specific Analysis

**Figure 6:**
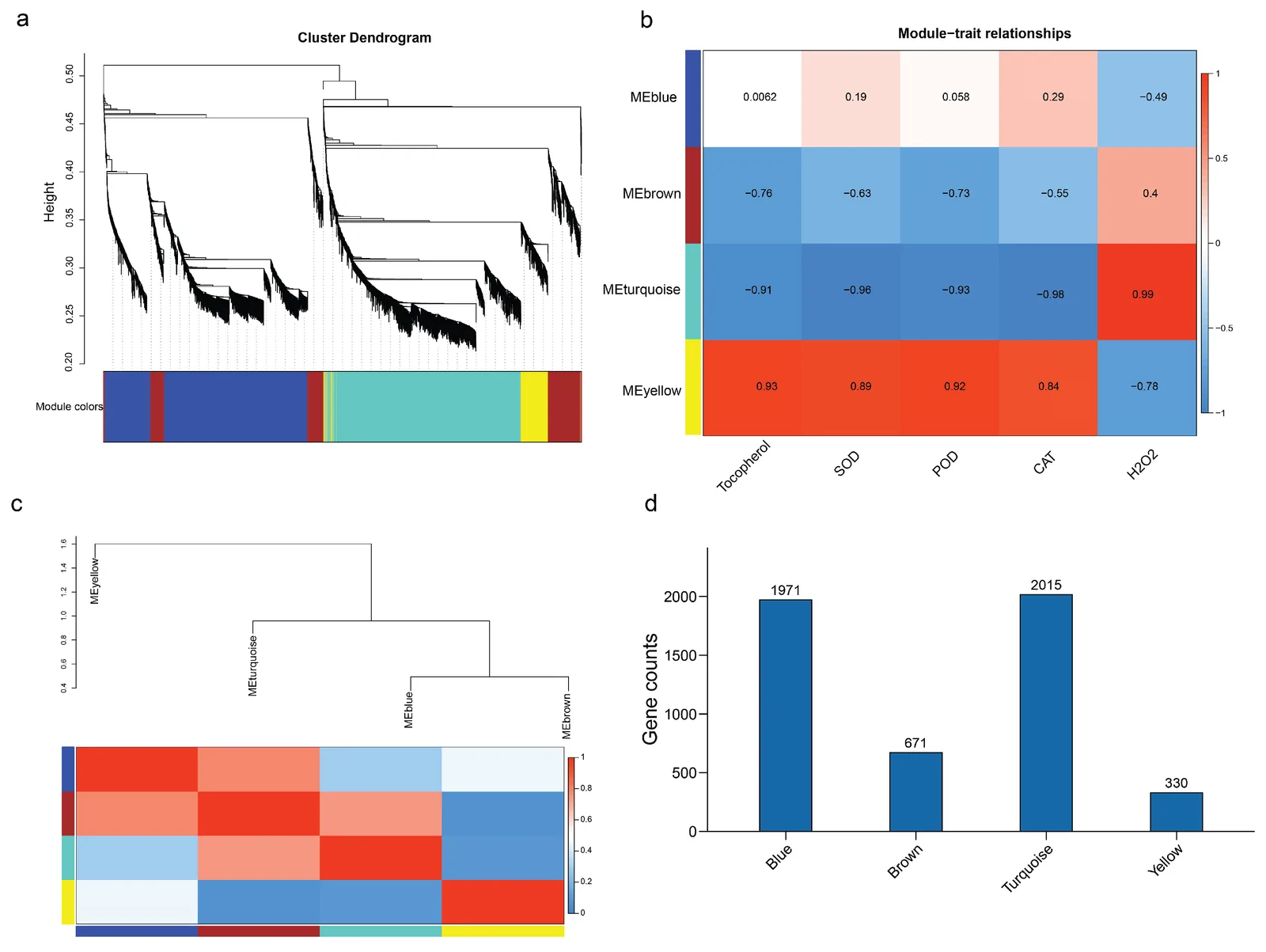
WGCNA trait-specific analysis of *BnVTE2* transgenic lines. (**a**) Cluster dendrogram showing four co-expression modules (blue, brown, turquoise, and yellow). (**b**) Module-trait correlation heatmap. (**c**) Module eigengene clustering dendrogram. (**d**) Bar plot showing the number of genes in each module.

To elucidate gene co-expression patterns associated with *BnVTE2* transgenic lines, Weighted Gene Co-expression Network Analysis (WGCNA) was conducted to identify modules of co-expressed genes and their relationships with physiological traits. Differentially expressed genes (DEGs) exhibiting minimal variation were excluded prior to network construction. A soft-thresholding power of β = 18 was selected to achieve scale-free topology (R^2^ ≈ 0.9), ensuring an appropriate balance between network sparsity and biological relevance (Figure S9). Hierarchical clustering identified four co-expression modules (Figure 6a). Module-trait analysis showed that the turquoise module correlated negatively with tocopherol, SOD, POD, and CAT and positively with H_2_O_2_ (r = 0.99). In contrast, the yellow module correlated positively with tocopherol (r = 0.93), SOD (r = 0.89), POD (r = 0.92), and CAT (r = 0.84) and negatively with H_2_O_2_ (r = −0.78). The brown module showed negative correlations with tocopherol, SOD, POD, and CAT and a positive correlation with H_2_O_2_ (r = 0.40), whereas the blue module showed weak correlations with all measured traits (|r| < 0.29) (Figure 6b). Module eigengene clustering showed distinct relationships among modules (Figure 6c). The blue, brown, turquoise, and yellow modules contained 1971, 671, 2015, and 330 genes, respectively (Figure 6d). These correlations identify the turquoise and yellow modules as candidate co-expression modules associated with tocopherol and antioxidant traits; they do not establish direct regulation by *BnVTE2*.

### 2.9. Detailed Analysis of BnVTE2-Associated Trait-Specific Modules

Further analysis focused on the turquoise and yellow modules, which showed strong correlations with measured antioxidant traits. Gene Ontology (GO) enrichment was used to summarize the functional annotations represented in each module. In the turquoise module, enriched biological-process terms included mitotic cell cycle, microtubule-based process, cell-cycle process, and microtubule-based movement; cellular-component terms included cytoskeleton, microtubule cytoskeleton, kinesin complex, and microtubule-associated complex; and molecular-function terms included thioglucosidase activity and ATP-dependent microtubule motor activity (Figure S10a). In the yellow module, enriched biological-process terms included anion transport, amino-acid transport, amino-acid transmembrane transport, and amino-acid import; cellular-component terms included plasma membrane, extracellular region, aleurone-grain membrane, and plant-type cell wall; and molecular-function terms included thioglucosidase activity and several transmembrane-transporter activities (Figure S10b).

**Figure 7:**
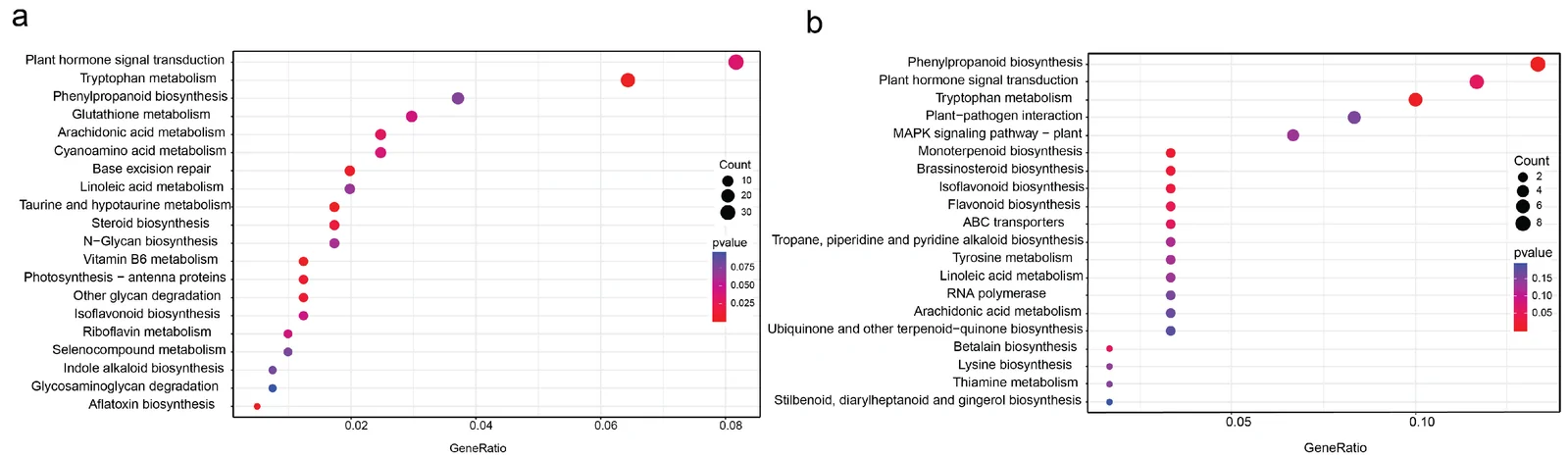

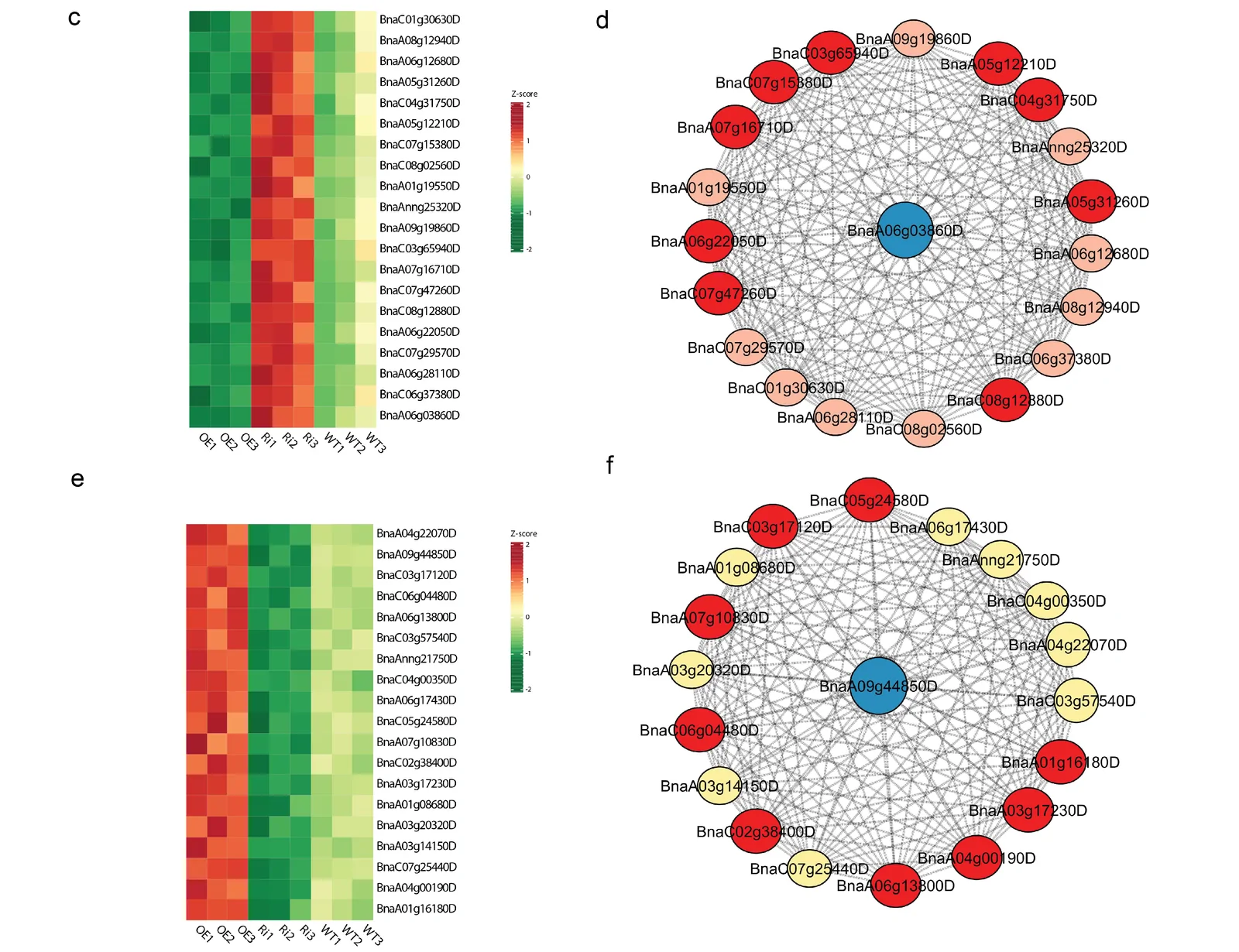
KEGG pathway and network analysis of the turquoise and yellow modules. (**a**) KEGG pathways enriched in the turquoise module. (**b**) KEGG pathways enriched in the yellow module. (**c**) Heatmap of expression profiles for the top 20 genes in the turquoise module, showing higher expression in RNAi lines. (**d**) Network of the top 10 hub genes in the turquoise module based on MCC and closeness, with node size and color reflecting connectivity. (**e**) Heatmap of expression profiles for the top 20 genes in the yellow module, showing higher expression in OE lines. (**f**) Network of the top 10 hub genes in the yellow module based on MCC and closeness, with node size and color reflecting connectivity.

KEGG enrichment of the turquoise module included glutathione metabolism, plant hormone signal transduction, phenylpropanoid biosynthesis, tryptophan metabolism, linoleic acid metabolism, aflatoxin biosynthesis, glycosaminoglycan degradation, indole alkaloid biosynthesis, and vitamin B6 metabolism (Figure 7a). The yellow module was enriched in phenylpropanoid biosynthesis, plant hormone signal transduction, tryptophan metabolism, linoleic acid metabolism, flavonoid biosynthesis, stilbenoid/diarylheptanoid/gingerol biosynthesis, ABC transporters, and brassinosteroid biosynthesis (Figure 7b). These non-lipid and signaling categories are reported as module-enrichment results and are not considered direct evidence of *BnVTE2*-controlled metabolic pathways.

Expression profiles of the top 20 genes in each module were compared across genotypes. In the turquoise module, most of these genes showed higher expression in RNAi lines, which also had higher H_2_O_2_ levels (Figure 7c). In the yellow module, several highly connected genes showed higher expression in OE lines, which had higher tocopherol, SOD, POD, and CAT values (Figure 7e). Network topology analysis ranked the top ten hub genes in each module using Maximum Clique Centrality (MCC) and closeness metrics (Figure 7d,f). These genes are therefore presented as candidate network hubs; their functional roles were not experimentally validated in this study.

## 3. Discussion

### 3.1. Vitamin E Levels Are Associated with Antioxidant Defense and Oxidative Status

VTE2 catalyzes the condensation of homogentisic acid with phytyl diphosphate to form MPBQ, a committed step in tocopherol biosynthesis. In this study, eight OE lines and eight RNAi lines were initially characterized, and selected homozygous T3 lines were used for detailed analyses. OE lines accumulated more total tocopherol than WT, whereas RNAi lines accumulated less (Figure S1c). This pattern is consistent with foundational Arabidopsis work establishing HPT/VTE2 as a major control point for tocopherol accumulation [3] and with recent *B. napus* analyses identifying *VTE2* expression as part of the developmental regulation of seed vitamin E accumulation [2].

Vitamin E is an important lipid-soluble, non-enzymatic antioxidant that limits lipid peroxidation and contributes to cellular redox homeostasis. In the present study, higher tocopherol accumulation in OE lines was accompanied by higher SOD, POD, and CAT activities and lower H_2_O_2_ accumulation, whereas RNAi lines showed lower tocopherol content and antioxidant enzyme activities together with higher H_2_O_2_ levels (Figure S1c and Figure 1a–d). These parallel changes indicate an association between tocopherol status and enzymatic antioxidant capacity but do not demonstrate that tocopherol directly regulates SOD, POD, or CAT. In this study, only enzyme activities were measured; we did not directly examine transcriptional regulation, protein abundance, enzyme activation, or physical interactions between tocopherol and these antioxidant enzymes. One possible explanation is that tocopherol, acting as a lipid-phase chain-breaking antioxidant, reduces lipid-peroxidation-derived radicals and the cellular ROS burden. The resulting changes in cellular redox status and ROS-dependent signaling could indirectly influence antioxidant enzyme activities. Conversely, reduced tocopherol accumulation in RNAi lines may create a more oxidizing cellular environment associated with impaired antioxidant capacity. These proposed relationships remain mechanistic hypotheses and require direct experimental validation.

### 3.2. Modulation of Fatty Acid Composition and Protection Against Lipid Peroxidation

Natural aging was used in this study to evaluate the association among *BnVTE2*-mediated tocopherol accumulation, fatty acid stability, lipid peroxidation, and seed longevity under prolonged storage conditions. Although accelerated aging assays are commonly used for rapid and controlled assessment of seed vigor, they impose acute high-temperature and high-humidity stress and may not fully represent the biochemical changes occurring during long-term seed storage. Therefore, the 42-month storage period provided a practical assessment of seed performance under natural aging conditions. However, because accelerated aging was not performed, future controlled deterioration assays would be valuable to further validate the contribution of *BnVTE2*-mediated tocopherol accumulation to seed longevity.

Although tocopherols do not participate directly in fatty-acid biosynthesis, genetic manipulation of *BnVTE2* was associated with shifts in relative fatty-acid profiles in *BnVTE2*-OE and RNAi lines. Fatty-acid synthesis occurs primarily in plastids, whereas triacylglycerol (TAG) assembly occurs predominantly in the endoplasmic reticulum (ER); therefore, the observed relationship between tocopherol status and TAG-related transcriptional changes should be interpreted as an indirect, cross-compartmental association rather than evidence of direct control of TAG assembly. Seeds with higher vitamin E content showed relatively lower proportions of oleic acid (C18:1) and linoleic acid (C18:2), but increased proportions of linolenic acid (C18:3), eicosanoic acid (C20:1), and dihomolinoleic acid (C20:2) (Figure 2). This shift may reflect an indirect association between vitamin E accumulation and fatty-acid availability in lipid assembly pathways [8]. After 42 months of storage, *BnVTE2*-RNAi seeds showed the greatest loss of polyunsaturated fatty acids, particularly C18:2 and C18:3, together with the highest MDA levels. These results suggest that reduced tocopherol accumulation may increase the susceptibility of polyunsaturated fatty acids to oxidative deterioration during prolonged storage.

Consistently, the positive association between tocopherol content and post-storage germination rate, with higher germination in OE lines and lower germination in RNAi lines, supports the view that endogenous tocopherols contribute to lipid stability and seed viability during long-term storage. This interpretation is consistent with foundational evidence from tocopherol-deficient Arabidopsis seeds [10] and with recent studies emphasizing lipid droplet and proteostasis mechanisms in seed longevity [11]. Nevertheless, the relationship between tocopherol accumulation and lipid protection should be interpreted with appropriate restraint. The natural-aging experiment provides phenotypic evidence that reduced tocopherol levels are associated with enhanced polyunsaturated fatty-acid degradation, lipid peroxidation, and reduced seed longevity. Unequivocal demonstration of causality will require future functional validation, such as genetic complementation, exogenous tocopherol application, targeted TAG and phospholipid profiling, and membrane lipid remodeling analysis.

### 3.3. Transcriptomic Reprogramming Links Vitamin E Levels to Core Metabolic Networks

Transcriptome profiling showed that *BnVTE2* modulation was accompanied by broad changes in gene expression. Because the central question of this study concerns tocopherol-associated lipid protection, interpretation was focused on lipid-related enrichments, including glycerolipid/glycerophospholipid metabolism and fatty-acid-related pathways. Phenylpropanoid biosynthesis and plant hormone signaling were also enriched, but these pathways were not supported by targeted metabolite, lignin, or hormone measurements in the present study and are therefore interpreted only as secondary transcriptional responses. Recent rapeseed studies similarly indicate that tocopherol accumulation is embedded within broader seed metabolic and developmental networks [1,2].

PPI and WGCNA analyses identified glycerolipid-related genes, including GPAT, TGL4, and GPD1, as candidate network hubs associated with *BnVTE2*-dependent expression differences (Figure 4). These network results are consistent with the observed changes in fatty-acid stability but do not establish direct regulation of lipid flux by tocopherols. Accordingly, glycerolipid metabolism is interpreted here as a candidate pathway linked to the *BnVTE2*/tocopherol phenotype. Targeted TAG and phospholipid profiling, membrane lipid analysis, and functional testing of hub genes will be required to determine whether these associations reflect causal metabolic remodeling.

### 3.4. Regulatory Role of Transcription Factors and Co-Expression Networks

Differential expression of AP2/ERF-ERF, NAC, and MYB transcription factors and the opposing WGCNA correlations of the yellow and turquoise modules indicate that *BnVTE2* manipulation is associated with broader transcriptional reprogramming (Figure 5 and Figure 6). The yellow module correlated positively with tocopherol and antioxidant enzyme activities, whereas the turquoise module correlated positively with H_2_O_2_. However, these correlations do not establish that tocopherols directly regulate these transcription factors or modules. We therefore consider the TF and co-expression results as candidate regulatory associations that should be tested using targeted expression, perturbation, and multi-omics experiments.

### 3.5. Limitations and Future Perspectives

Although this study provides *Brassica napus*-specific evidence linking *BnVTE2*-mediated tocopherol accumulation with antioxidant capacity, fatty-acid stability, and seed longevity, several limitations should be considered. First, the natural-aging experiment reflects prolonged storage under ordinary laboratory conditions but is less tightly controlled than accelerated-aging or controlled-deterioration assays. The seeds were stored at room temperature, and exact temperature and relative humidity were not continuously recorded; environmental variation may therefore have contributed to the aging response. Second, fatty-acid values were calculated by chromatographic peak-area normalization and represent relative composition rather than absolute fatty-acid quantities. Changes in percentages should not be interpreted as direct measurements of fatty-acid synthesis, degradation, or total lipid content. Third, transcriptomic, PPI, and WGCNA analyses identify correlations, enrichments, and candidate networks rather than causal regulatory relationships. The predicted glycerolipid-related hubs were not validated by targeted gene-function assays, TAG quantification, phospholipid profiling, or membrane lipidomics. Accordingly, glycerolipid metabolism is considered a candidate pathway associated with *BnVTE2*-mediated changes rather than an experimentally confirmed target of direct regulation. Future work should combine controlled aging assays with absolute lipid quantification, targeted lipidomics, genetic complementation or tocopherol-rescue experiments, and functional analysis of candidate hub genes such as *GPAT*, *GPD1*, *TGL4*, and *ACOX1*.

## 4. Materials and Methods

### 4.1. Plants

*Brassica napus* cv. ‘Westar’ was used to generate *BnVTE2*-OE and *BnVTE2*-RNAi lines. The full *BnVTE2* coding sequence and a *BnVTE2*-specific fragment corresponding to nucleotides 605–850 of the coding sequence were amplified from Westar cDNA and cloned into pENTR (ThermoFisher Scientific, Shanghai, China). After sequence verification, the full coding sequence and RNAi fragment were transferred by LR recombination into pH7WG2 and pWatergate, respectively. The constructs were introduced into Westar by Agrobacterium tumefaciens-mediated transformation using strain GV3101::pMP90RK. Regenerated transformants were confirmed by PCR using the primers listed in Table S1, and *BnVTE2* expression was quantified by RT-qPCR. Independent lines were advanced by self-pollination, and third-generation (T3) homozygous OE and RNAi plants were used for downstream analyses; WT plants were cultivated in parallel. During early cultivation, transgenic and WT plants were maintained under the same conditions and, after establishment, were transferred to the field, where all genotypes were grown concurrently under the same field conditions. Plants were bagged at flowering to minimize cross-pollination among lines. Developing siliques were sampled at 20, 30, and 40 days after pollination (DAP) and stored at −80 °C until analysis. Agronomic productivity/yield was not quantitatively assessed in this study.

### 4.2. Physiological and Biochemical Analysis

Vitamin E content was determined using a Vitamin E (VE) Content Assay Kit (BC1420, Solarbio Life Sciences, Beijing, China) according to the manufacturer’s instructions. Briefly, approximately 0.1 g of seed tissue was homogenized in 0.2 mL ddH_2_O, 0.3 mL ethanol, and 0.5 mL n-heptane. After incubation for 5 min at 25 °C and centrifugation at 5000× *g* for 5 min, the upper n-heptane phase was collected and mixed with 0.9 mL ethanol. Vitamin E content was calculated from a standard curve generated from the absorbance of vitamin E standards at 510 nm.

Hydrogen peroxide (H_2_O_2_) content was determined using assay kit G0112F (Grace Biotechnology, Suzhou, Jiangsu, China). Approximately 0.1 g of seed tissue was homogenized in 1 mL acetone, centrifuged at 12,000 rpm and 4 °C for 10 min, and the supernatant was collected for analysis. H_2_O_2_ content was calculated from absorbance measured at 415 nm with a SpectraMax iD5 microplate reader (Molecular Devices, San Jose, CA, USA). Malondialdehyde (MDA) content in seedlings was determined using assay kit S0131S (Beyotime Biotechnology, Shanghai, China) according to the manufacturer’s instructions.

SOD, POD, and CAT activities were determined using activity assay kits G0104F, G0317F, and G0105F, respectively (Grace Biotechnology), according to the manufacturer’s instructions. Approximately 0.1 g of seed tissue was homogenized in 1 mL extraction buffer and centrifuged at 12,000 rpm and 4 °C for 10 min. The supernatant was used for the assays. SOD, POD, and CAT activities were determined from absorbance at 450, 470, and 510 nm, respectively.

### 4.3. Fatty Acid Composition in Seeds

Fatty-acid composition was analyzed by gas chromatography-mass spectrometry (GC-MS) using mass-selective detection (MSD) (Agilent Technologies, Santa Clara, CA, USA) after conversion of seed lipids to fatty-acid methyl esters (FAMEs). For each biological replicate, 3–5 seeds were homogenized in a microcentrifuge tube and extracted with ether:petroleum ether (1:1, *v*/*v*) by shaking for 10 min followed by incubation for 40 min. KOH in methanol (500 μL) and 20 μg of C17:0 internal standard (TMstandard^®^) were added, and methylation proceeded for 30 min. The reaction was stopped with 500 μL ddH_2_O, and the phases were separated by centrifugation. FAMEs were separated on a DB-FastFAME capillary column (Agilent; 30 m × 0.25 mm i.d., 0.25 μm film thickness). The total chromatographic run time was 13 min. Individual fatty acids were identified using retention times of commercial standards together with their mass-spectral profiles (Figure S11), and relative fatty-acid composition (%) was calculated by chromatographic peak-area normalization. Three biological replicates were analyzed for each line.

### 4.4. Seed Storage and Natural-Aging Assay

Mature seeds of WT, *BnVTE2*-OE, and *BnVTE2*-RNAi lines were harvested at physiological maturity, air-dried, and stored for 6 or 42 months under the same natural-aging conditions. Seeds were kept in unsealed paper seed bags in darkness at room temperature under ambient laboratory air. All genotypes and biological replicates were stored in the same room under identical conditions. No deliberate oxygen restriction, vacuum sealing, or inert-gas flushing was applied.

Natural aging was selected because the objective of this study was to evaluate seed viability and lipid peroxidation after prolonged storage. Accelerated aging assays are useful for rapid evaluation of seed vigor under controlled deterioration conditions; however, they impose acute high-temperature and high-humidity stress and may not fully represent biochemical changes occurring during long-term storage. Therefore, the 42-month natural-aging assay was used to assess seed storability under prolonged storage conditions.

### 4.5. RNA Extraction and Transcriptome Sequencing

Total RNA was isolated from developing rapeseed seeds collected at 30 days after pollination (DAP) using TRIzol reagent (Invitrogen, Carlsbad, CA, USA). RNA purity and concentration were assessed using a NanoDrop 2000 spectrophotometer (Thermo Scientific, Waltham, MA, USA) and an Agilent 2100 Bioanalyzer (Agilent Technologies, Palo Alto, CA, USA). RNA-seq libraries were constructed using the Illumina TruSeq RNA Sample Preparation Kit (Illumina, San Diego, CA, USA) according to the manufacturer’s protocol. Indexed libraries were pooled and sequenced in a 2 × 150 bp paired-end configuration on an I Illumina NovaSeq platform.

### 4.6. Transcriptome Data Processing and Analysis

Raw sequencing reads were processed with Cutadapt (v1.9.1) to remove adapters, PCR-primer sequences, and low-quality bases (Phred score < 20), using an error rate of 0.1, a minimum adapter overlap of 1 bp, a minimum read length of 75 bp, and a maximum ambiguous-base (N) proportion of 0.1. Clean reads were mapped to the *Brassica napus* reference genome obtained from BrassicaDB using HISAT2 [12]. Gene expression was quantified across 9 libraries as fragments per kilobase of transcript per million mapped fragments (FPKM). Differentially expressed genes (DEGs) were identified using edgeR with thresholds of |log_2_FC| > 2 and FDR-adjusted *p* ≤ 0.05 [13].

Gene Ontology (GO) enrichment analysis was performed using GOSeq (version 1.34.1) to annotate DEGs with statistically significant enrichment (*p*adj ≤ 0.05) [14]. Additionally, Kyoto Encyclopedia of Genes and Genomes (KEGG) pathway enrichment was conducted using custom scripts to identify significantly enriched pathways among DEGs [15].

### 4.7. Construction and Analysis of Protein-Protein Interaction (PPI) Network

To investigate the potential interactions among differentially expressed genes (DEGs) across the selected metabolic pathways, a protein-protein interaction (PPI) network was constructed using the STRING database (v12.0) [16]. The *Brassica napus* protein sequences corresponding to DEGs were queried in STRING with a medium confidence score cutoff of 0.4 to ensure biologically meaningful interactions. The resulting interaction network was downloaded in TSV format and visualized using Cytoscape software (v3.10.0) [17].

Network topological analysis and hub gene identification were performed using the cytoHubba plugin implemented in Cytoscape [18]. The Maximal Clique Centrality (MCC) algorithm was employed to rank nodes (genes) based on their network connectivity and importance. The top 10-ranked genes were considered as hub genes.

### 4.8. Identification and Analysis of Transcription Factors (TFs)

To identify transcription factors (TFs) responsive to *BnVTE2* modulation, differentially expressed genes (DEGs) obtained from the transcriptomic analysis of the three genotypes (OE, RNAi, and WT) were subjected to TF family classification. The DEG lists from each pairwise comparison (OE-vs-WT, RNAi-vs-WT, and OE-vs-RNAi) were annotated based on sequence homology to known TFs in the Plant Transcription Factor Database (PlantTFDB, v4.0) [19]. TF prediction and classification were performed using the BLASTP algorithm with an E-value threshold of 1 × 10^−5^, minimum sequence identity of 50%, and alignment coverage ≥ 70%. Each DEG was assigned to a specific TF family according to the annotation in PlantTFDB.

### 4.9. Weighted Gene Co-Expression Network Analysis (WGCNA)

Weighted Gene Co-expression Network Analysis (WGCNA) was performed using the R package WGCNA to identify gene modules associated with physiological traits in *BnVTE2* transgenic lines. Normalized expression values of DEGs were used after filtering low-variance genes. A soft-thresholding power (β = 18) was chosen to achieve scale-free topology (R^2^ ≈ 0.9) [20]. Genes were clustered using the dynamic tree cut method, and modules were defined with a minimum size of 30 genes. Module trait correlations were computed using Pearson’s correlation between module eigengenes and traits. Modules showing significant correlations (|r| ≥ 0.7, *p* < 0.05) were selected for enrichment and hub gene analysis. GO and KEGG enrichment analyses were conducted using clusterProfiler with an FDR < 0.05 [21]. Co-expression networks of significant modules were visualized in Cytoscape, and hub genes were identified using the cytoHubba plugin based on Maximal Clique Centrality (MCC) [17,18]. The top 10 ranked genes from each module were designated as hub genes.

### 4.10. Statistical Analysis

All statistical analyses were performed using GraphPad Prism 9 (GraphPad Software, San Diego, CA, USA). Quantitative data are presented as mean ± standard deviation (SD) from the biological replicates indicated for each experiment. Differences among WT, OE, and RNAi lines were evaluated using analysis of variance (ANOVA), followed by appropriate multiple-comparison tests when significant differences were detected. When the assumption of homogeneity of variance was not satisfied, Welch’s one-way ANOVA followed by Games Howell multiple comparisons was used. For experiments conducted at different developmental stages or storage durations, comparisons among genotypes were performed at the corresponding time points. A two-sided *p* value < 0.05 was considered statistically significant.

## 5. Conclusions

This study shows that modulation of *BnVTE2* expression in *Brassica napus* alters tocopherol accumulation and is associated with changes in antioxidant enzyme activity, ROS/MDA levels, fatty acid stability, lipid-related gene expression, and seed performance after long-term storage. Transcriptomic and network analyses identified glycerolipid-related genes as candidate hubs potentially linked to these responses. However, because TAG content, phospholipid composition, membrane lipid remodeling, and direct hub-gene functions were not experimentally validated, these pathways should be considered candidate mechanisms for future study. Overall, our findings support an association among *BnVTE2*-mediated tocopherol accumulation, oxidative protection, and seed longevity in *B. napus*.

## Data Availability

The raw transcriptome data generated in this study will be made available by the authors upon reasonable request.

## Supplementary Materials

The following supporting information can be downloaded at: https://www.mdpi.com/article/10.3390/plants15182757/s1, Table S1: Primers used in the study; Table S2: Selected genes in pathways associated with *BnVTE2* modulation; Table S3: RNA-seq mapping statistics for OE, RNAi, and WT libraries; Table S4: Top 10 hub genes in the *BnVTE2* lipid PPI network; Figure S1: *BnVTE2* expression and tocopherol accumulation in transgenic *Brassica napus* lines; Figure S2: Quality control and Gene Ontology (GO) enrichment analysis; Figure S3: Schematic representation of the phenylpropanoid biosynthesis pathway, illustrating the conversion of phenylalanine and tyrosine into various phenolic compounds and lignins; Figure S4: Schematic representation of the α-linolenic acid metabolism pathway, detailing the conversion of α-linolenic acid and its derivatives into various compounds, including volatile organic compounds and jasmonates; Figure S5: Schematic representation of the linoleic acid metabolism pathway, depicting the conversion of linoleate into various hydroperoxides, epoxides, and other derivatives; Figure S6: Schematic representation of the glycerophospholipid metabolism pathway, illustrating the conversion of glycerol-3-phosphate and related inter-mediates into various phospholipids and lysophospholipids; Figure S7: Schematic representation of the glycerolipid metabolism pathway, illustrating the conversion of glycerol-3-phosphate and related intermediates into various glycerolipids, including monoacylglycerol, diacylglycerol, and triacylglycerol; Figure S8: Schematic representation of the fatty acid degradation pathway, illustrating the breakdown of hexadecanoyl-CoA into acetyl-CoA and other short-chain acyl-CoAs through β-oxidation; Figure S9: Analysis of scale independence and mean connectivity for network construction; Figure S10: Gene Ontology (GO) enrichment analysis of genes in WGCNA modules; Figure S11: GC-MS total ion chromatograms (TICs) and mass spectral identification of commercial fatty-acid methyl ester standards and wild-type rapeseed.

## Abbreviations

CAT, catalase; DAP, days after pollination; DEG, differentially expressed gene; FAME, fatty-acid methyl ester; FDR, false discovery rate; FPKM, fragments per kilobase of transcript per million mapped fragments; GC-MS, gas chromatography-mass spectrometry; GO, Gene Ontology; H_2_O_2_, hydrogen peroxide; KEGG, Kyoto Encyclopedia of Genes and Genomes; MCC, Maximal Clique Centrality; MDA, malondialdehyde; MSD, mass-selective detection; OE, overexpression; PCA, principal component analysis; POD, peroxidase; PPI, protein-protein interaction; RNAi, RNA interference; RNA-seq, RNA sequencing; ROS, reactive oxygen species; SOD, superoxide dismutase; TAG, triacylglycerol; TF, transcription factor; WGCNA, weighted gene co-expression network analysis; WT, wild type.
