# Supplementary material for: BnVTE2-Mediated Vitamin E Modulation Is Associated with Antioxidant Capacity, Fatty Acid Stability, and Seed Longevity in Brassica napus
Source: Plants (Basel). 2026 Sep 9;15(18):2757. doi: 10.3390/plants15182757 (PMC13610400; doi:10.3390/plants15182757)
Supplement: Supplementary file 1 [file plants-15-02757-s001.zip › plants-4464892-supplementary.pdf]

Title: *BnVTE2*-Mediated Vitamin E Modulation Is Associated with Antioxidant Capacity, Fatty Acid Stability, and Seed Longevity in *Brassica napus*

Qiao Ruan 1, Xiaoli Tan 1, Wajahat Hussain 2, Yunxia Zeng 2 and Yonghong Zhou 2,*

1 Chongqing Chemical Industry Vocational College, Putidong Road 2009, Changshou District, Chongqing 401228, China; qiaoruanchemistry@outlook.com (Q.R.); tanxl023@outlook.com (X.T.)

2 College of Agronomy and Biotechnology, Southwest University, Tiansheng Road 2, Beibei District, Chongqing 400715, China; wajahat7253@yahoo.com (W.H.); zyx071316@email.swu.edu.cn (Y.Z.)

* Correspondence: zhou2017@swu.edu.cn; Tel.: +86-177-2303-4767

Supplementary Information

Table S1. Primers used in the study.

Primer	Sequence (5′–3′)	Description
YZ6	CACCATGGAGTCTCTCCTCTCTAGTTC	To clone *BnVTE2* CDS
YZ7	TCACTTCAGAAAAGGTAACAGC	To clone *BnVTE2* CDS
YZ25	CAAGCTAAGCTTGAGCTCTCCC	35S_F
YZ26	TGCAGGTCGACTAGAATAGTAAATTG	35S_R
YZ27	CACCTTGGTTCATGGCCATTGTTCTGG	To clone *BnVTE2* specific tag
YZ28	CAGAGAAGAAGCTCATAAACGC	To clone *BnVTE2* specific tag
YZ429	TGAAGATCAAGGTGGTCGCA	for *BnActin7* qPCR
YZ430	AGAAGGCAGAAACACTTAGAAG	for *BnActin7* qPCR
YZ445	GCGTTTATGAGCTTCTTCTCTG	for *BnVTE2* qPCR
YZ446	CAGCGTAAGCCATTTGAAGTAG	for *BnVTE2* qPCR

Table S2. Selected genes in pathways associated with *BnVTE2* modulation.

A. Phenylpropanoid Biosynthesis

No.	Gene Name	Reg.	Log_2FC (OE vs WT)	FDR (OE vs WT)	Log_2FC (RNAi vs WT)	FDR (RNAi vs WT)	Function
1	*BnaC02g10690D*	↓B	-5.264	7.51×10^{-24}	-4.368	1.42×10^{-22}	Peroxidase (ROS scavenging)
2	*BnaC01g28040D*	↓B	-3.550	1.13×10^{-9}	-3.165	4.31×10^{-10}	Peroxidase
3	*BnaC02g18660D*	↓B	-2.944	2.90×10^{-17}	-1.339	5.40×10^{-7}	Peroxidase
4	*BnaA02g14050D*	↓B	-2.334	3.36×10^{-74}	-1.305	5.92×10^{-18}	Peroxidase
5	*BnaCnng30570D*	↑B	2.320	1.19×10^{-7}	2.111	1.74×10^{-5}	HCT (phenylpropanoid metabolism)
6	*BnaC02g13760D*	↓O	-1.737	2.24×10^{-4}	-0.629	0.187	COMT (lignin methylation)
7	*BnaA03g32150D*	↓B	-1.259	5.89×10^{-21}	-2.139	1.20×10^{-52}	SNG2 (sinapate modification)
8	*BnaC07g42360D*	↑O	2.384	3.48×10^{-56}	0.269	0.35	Peroxidase
9	*BnaAnng10890D*	↑O	2.295	1.85×10^{-48}	-0.047	0.922	Peroxidase
10	*BnaA01g18650D*	↑R	0.914	1.22×10^{-9}	1.087	6.76×10^{-15}	HCT

B. α-Linolenic Acid Metabolism

No.	Gene Name	Reg.	Log_2FC (OE vs WT)	FDR (OE vs WT)	Log_2FC (RNAi vs WT)	FDR (RNAi vs WT)	Function
1	*BnaAnng32170D*	↑R	0.572	0.0654	5.439	4.84×10^{-225}	OPCL1 (JA synthesis)
2	*BnaA07g21050D*	↓B	-1.403	3.17×10^{-6}	-4.982	4.78×10^{-51}	ADH1 (JA pathway)
3	*BnaC06g21150D*	↓B	-1.910	1.40×10^{-8}	-5.604	1.53×10^{-50}	ADH1
4	*BnaA09g19570D*	↑O	1.091	7.41×10^{-6}	0.638	0.0106	AOC (JA synthesis)

No.	Gene Name	Reg.	Log2FC (OE vs WT)	FDR (OE vs WT)	Log2FC (RNAi vs WT)	FDR (RNAi vs WT)	Function
5	*BnaC09g52550D*	↑O	1.629	8.63×10^{-12}	0.960	1.13×10^{-4}	AOC
6	*BnaC07g22930D*	↑O	1.701	1.23×10^{-8}	0.122	0.867	AOC
7	*BnaC06g23490D*	NS	0.826	1.51×10^{-6}	-0.385	0.0866	DOX (lipid oxidation)
8	*BnaC05g46590D*	↑O	1.045	0.0015	0.358	0.299	DOX
9	*BnaA07g11420D*	↓O	-1.045	0.0143	-0.488	0.201	OPCL1
10	*BnaA06g13800D*	↑O/↓R	1.532	1.01×10^{-12}	-1.559	8.72×10^{-6}	JMT (JA methylation)

C. Linoleic Acid Metabolism

No.	Gene Name	Reg.	Log2FC (OE vs WT)	FDR (OE vs WT)	Log2FC (RNAi vs WT)	FDR (RNAi vs WT)	Function
1	*BnaAnng31420D*	↓R	0.162	0.618	-1.307	2.76×10^{-10}	CYP81 (linoleic hydroxylation)
2	*BnaC03g33430D*	↓R	-0.002	1	-1.291	2.40×10^{-8}	CYP89 (fatty acid oxidation)
3	*BnaC06g42070D*	NS	0.718	3.82×10^{-8}	-0.726	5.78×10^{-7}	CYP81
4	*BnaCnng62240D*	NS	0.565	3.20×10^{-5}	-0.681	7.32×10^{-6}	CYP71 (linoleic metabolism)
5	*BnaC05g30190D*	↓O	-1.210	3.34×10^{-21}	-0.654	1.08×10^{-5}	LOX1 (linoleate 9S-lipoxygenase)
6	*BnaA01g21450D*	↓B	-1.536	4.21×10^{-16}	-1.224	1.35×10^{-10}	LOX1
7	*BnaA05g19020D*	↑B	2.048	1.56×10^{-30}	1.648	9.12×10^{-17}	CYP94 (linoleic epoxidation)
8	*BnaA01g18990D*	NS	0.563	0.0189	-0.829	0.0014	CYP71
9	*BnaA02g29390D*	↓R	0.950	10.00×10^{-5}	-1.824	2.24×10^{-8}	CYP81
10	*BnaA05g12840D*	NS	0.663	8.94×10^{-6}	-0.668	1.37×10^{-4}	CYP94

D. Glycerophospholipid Metabolism

No.	Gene Name	Reg.	Log₂FC (OE vs WT)	FDR (OE vs WT)	Log₂FC (RNAi vs WT)	FDR (RNAi vs WT)	Function
1	*BnaA09g44850D*	↑O/↓R	1.165	2.58×10^{-18}	-1.691	2.14×10^{-14}	Phosphoethanolamine phosphatase
2	*BnaC08g01840D*	NS	-0.695	0.004	0.329	0.147	3-hydroxyisobutyryl-CoA hydrolase
3	*BnaC03g78050D*	↓O/↑R	-1.577	3.34×10^{-4}	1.698	1.08×10^{-13}	Lysophospholipase II
4	*BnaAnng30500D*	↓R	0.366	0.0671	-1.612	1.38×10^{-14}	PHOSPHO1 (choline recycling)
5	*BnaA07g30540D*	↓B	-1.551	4.51×10^{-14}	-1.223	8.15×10^{-10}	PHOSPHO1
6	*BnaA01g28540D*	↓R	-0.185	0.603	-1.144	1.07×10^{-6}	Phospholipase D1
7	*BnaC08g37550D*	↑O/↓R	1.661	3.09×10^{-7}	-2.079	3.23×10^{-4}	PHOSPHO1
8	*BnaC08g17490D*	↓R	0.243	0.284	-1.302	4.74×10^{-11}	PHOSPHO1
9	*BnaCnng71810D*	↓R	-0.119	0.845	-1.545	2.85×10^{-6}	Glutamate decarboxylase
10	*BnaC03g25130D*	↓R	-0.663	0.0015	-1.042	5.39×10^{-7}	Phosphatidate cytidylyltransferase

E. Glycerolipid Metabolism

No.	Gene Name	Reg.	Log₂FC (OE vs WT)	FDR (OE vs WT)	Log₂FC (RNAi vs WT)	FDR (RNAi vs WT)	Function
1	*BnaCnng52810D*	NS	0.172	0.383	-0.915	1.10×10^{-10}	Diacylglycerol O-acyltransferase
2	*BnaCnng02250D*	↑O	1.127	6.61×10^{-16}	0.115	0.648	Digalactosyldiacylglycerol synthase
3	*BnaC08g27260D*	NS	0.214	0.187	-0.806	4.55×10^{-7}	Alpha-galactosidase
4	*BnaC04g53550D*	NS	0.434	0.495	-0.993	1.37×10^{-5}	Alcohol dehydrogenase (NADP+)

No.	Gene Name	Reg.	Log2FC (OE vs WT)	FDR (OE vs WT)	Log2FC (RNAi vs WT)	FDR (RNAi vs WT)	Function
5	*BnaA02g33390D*	↑O	1.002	6.94×10^{-5}	-0.343	0.65	AKR1
6	*BnaA08g20300D*	↓B	-1.426	9.39×10^{-18}	-1.180	6.99×10^{-11}	Aldehyde dehydrogenase
7	*BnaA08g04430D*	↓B	-1.340	1.73×10^{-15}	-1.402	8.28×10^{-19}	ALDH
8	*BnaA02g19720D*	↓B	-1.526	3.29×10^{-14}	-1.035	1.49×10^{-8}	Glycerol kinase
9	*BnaA07g35620D*	↓B	-1.543	2.85×10^{-14}	-1.550	2.67×10^{-9}	Glycerol kinase
10	*BnaC06g19150D*	↓R	-0.535	0.041	-1.177	5.86×10^{-7}	D-glycerate 3-kinase

F. Fatty Acid Degradation

No.	Gene Name	Reg.	Log2FC (OE vs WT)	FDR (OE vs WT)	Log2FC (RNAi vs WT)	FDR (RNAi vs WT)	Function
1	*BnaA07g21050D*	↓B	-1.403	3.17×10^{-6}	-4.982	4.78×10^{-51}	Alcohol dehydrogenase
2	*BnaC06g21150D*	↓B	-1.910	1.40×10^{-8}	-5.604	1.53×10^{-50}	ADH1 (peroxisomal detox)
3	*BnaA05g16170D*	↓B	-1.255	6.26×10^{-17}	-1.155	2.65×10^{-14}	Long-chain acyl-CoA synthetase
4	*BnaA04g06040D*	↓B	-1.237	8.72×10^{-9}	-1.222	1.06×10^{-10}	Enoyl-CoA isomerase
5	*BnaC05g51350D*	↓B	-1.364	4.30×10^{-22}	-1.375	1.90×10^{-24}	ACSL
6	*BnaA08g04430D*	↓B	-1.340	1.73×10^{-15}	-1.402	8.28×10^{-19}	Aldehyde dehydrogenase
7	*BnaA08g20300D*	↓B	-1.426	9.39×10^{-18}	-1.180	6.99×10^{-11}	ALDH
8	*BnaA04g01830D*	↑O	1.129	1.49×10^{-6}	0.512	0.0623	Citrate synthase
9	*BnaA05g31340D*	↑R	0.576	0.134	1.584	1.59×10^{-9}	ACSL
10	*BnaC08g05150D*	↓B	-3.063	2.85×10^{-35}	-2.331	3.21×10^{-29}	ALDH

Table S3. RNA-seq mapping statistics for OE, RNAi, and WT libraries.

Sample	Total Reads	mapped Reads	map Rate	uniq	paired	single	Self ANDmate	mapDiffCHR
OE1	49707652	45903362	92.35 %	42007207	40366054	1822716	44080646	429682
OE2	58083096	53686844	92.43 %	48922532	47008332	2135226	51551618	518824
OE3	62644828	57606121	91.96 %	52495417	50411466	2314595	55291526	596856
Ri1	52361916	47504227	90.72 %	44020243	42207596	1983379	45520848	421436
Ri2	61775448	56328926	91.18 %	52079721	49974396	2303792	54025134	525664
Ri3	57335002	52206765	91.06 %	48316319	46291492	2214467	49992298	507544
WT1	56534350	51842664	91.70 %	47738638	45847188	2080932	49761732	473164
WT2	56495014	51776644	91.65 %	47737298	45823174	2103858	49672786	473680
WT3	49409750	45370198	91.82 %	41754904	40090558	1823034	43547164	422516

Table S4. Top 10 hub genes in the *BnVTE2* lipid PPI network.

Rank	Gene name	Pathway	Degree	Log_2FC (OE)	Log_2FC (RNAi)	Function
1	*BnaC02g02780D*	GL metabolism	14	-0.78	-0.35	TGL4 (TAG lipase)
2	*BnaCnng10420D*	GL metabolism	14	-0.97	-0.2	TGL4 (TAG lipase)
3	*BnaA09g00450D*	GL metabolism	8	1.1	0.4	GPAT (G3P acyltransferase)
4	*BnaAnng31310D*	GL metabolism	8	-4.5	-2.8	GPAT (G3P acyltransferase)
5	*BnaCnng74890D*	GL metabolism	8	-2.8	-2.5	GPAT (G3P acyltransferase)
6	*BnaC05g04490D*	GL metabolism	6	-0.35	-0.35	GPAT (G3P acyltransferase)
7	*BnaA04g10460D*	GL metabolism	5	-0.7	-1	GPD1 (G3P dehydrogenase)
8	*BnaC04g01960D*	GL metabolism	5	-3.2	-2.8	GPD1 (G3P dehydrogenase)
9	*BnaC04g32730D*	GL metabolism	5	0.5	-0.2	GPD1 (G3P dehydrogenase)
10	*BnaA09g07080D*	FA degradation	14	-1.2	-0.7	ACOX1 (Fatty acid oxidation)

Note: Hubs ranked by MCC (cytoHubba). Degree = connections. Log_2FC calculated from raw CPM data.

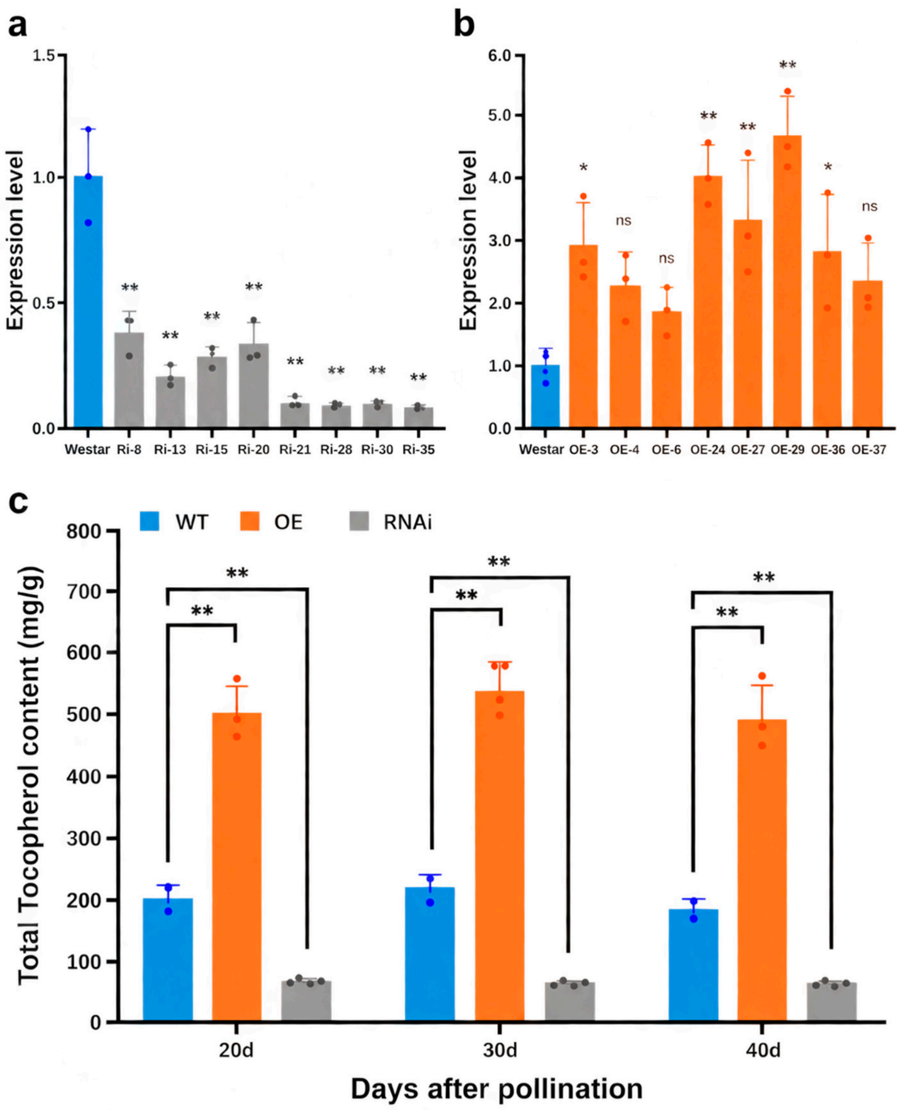

Figure S1. BnVTE2 expression and tocopherol accumulation in transgenic Brassica napus lines. (a) Relative BnVTE2 expression in leaves of independent RNAi lines. (b) Relative BnVTE2 expression in leaves of independent OE lines. (c) Total tocopherol content in developing seeds at 20, 30, and 40 days after pollination. Asterisks indicate significant differences as defined in the statistical analysis (* $p < 0.05$; ** $p < 0.01$); ns, not significant.

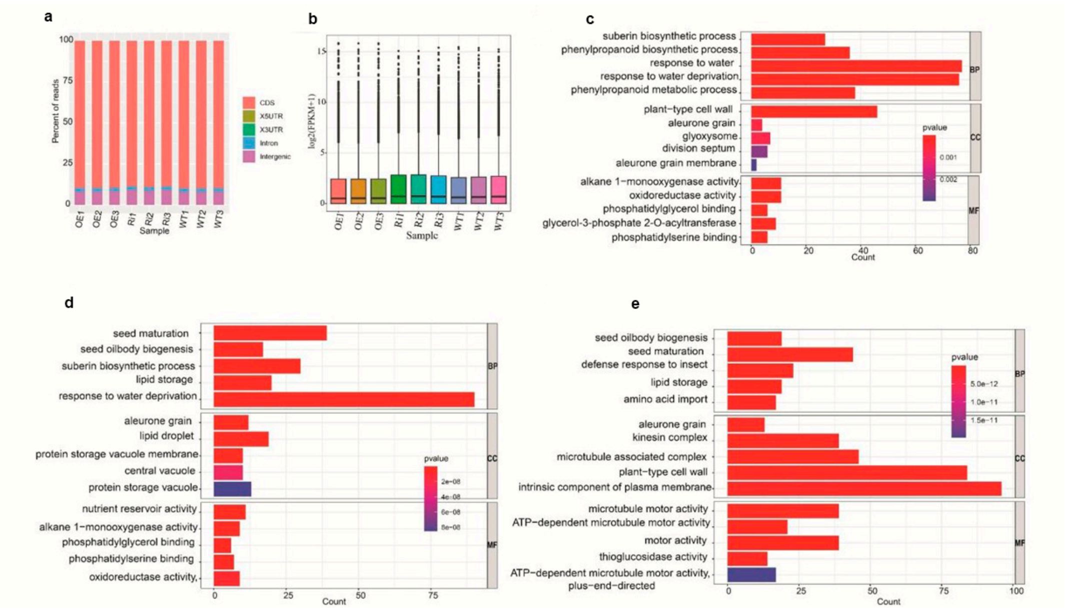

Figure S2. Quality control and Gene Ontology (GO) enrichment analysis. (a) Stacked bar plot showing the proportions of reads mapped to genomic features across samples. (b) Boxplots showing the $\log_2$(FPKM + 1) expression distribution across all samples. (c) GO enrichment analysis for OE vs. WT. (d) GO enrichment analysis for RNAi vs. WT. (e) GO enrichment analysis for OE vs. RNAi.

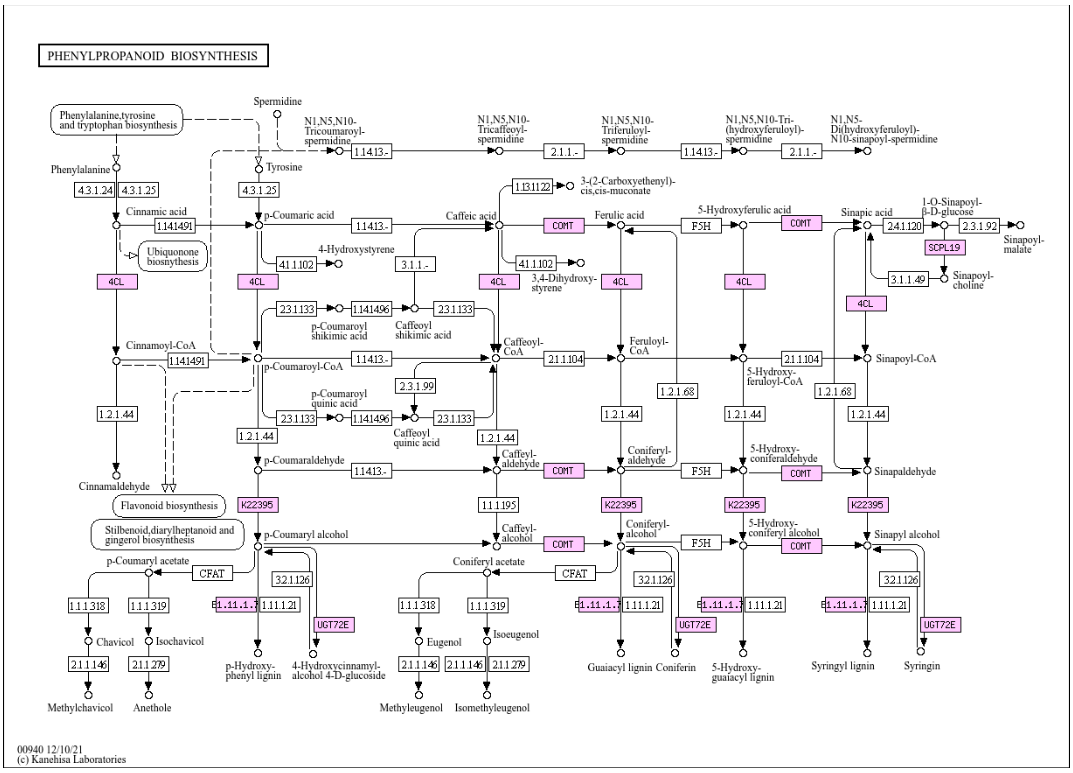

Figure S3. Schematic representation of the phenylpropanoid biosynthesis pathway, illustrating the conversion of phenylalanine and tyrosine into various phenolic compounds and lignins. Colored highlights indicate changes in the pathway.

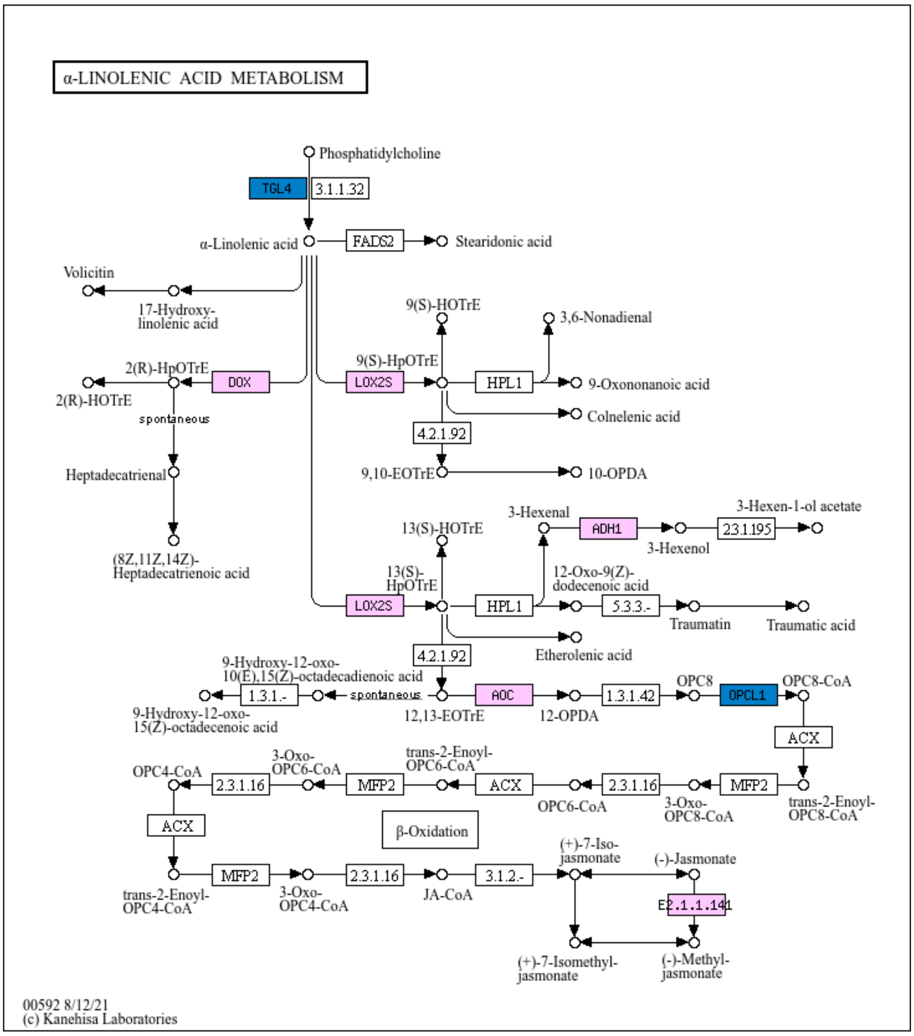

Figure S4. Schematic representation of the α-linolenic acid metabolism pathway, detailing the conversion of α-linolenic acid and its derivatives into various compounds, including volatile organic compounds and jasmonates. Colored highlights indicate changes in the pathway.

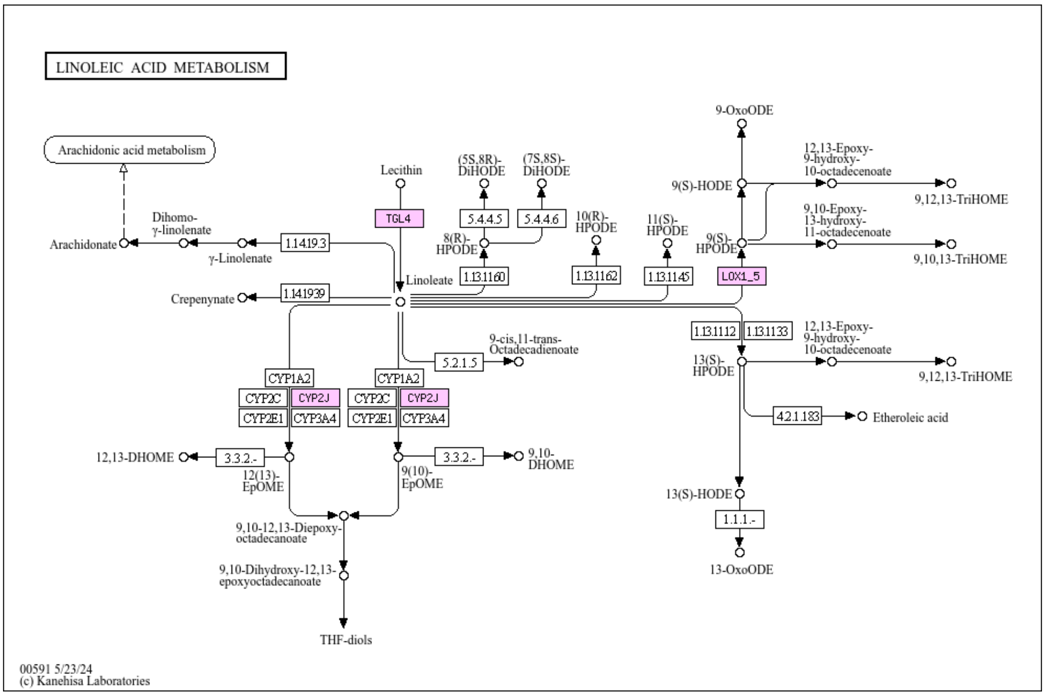

Figure S5. Schematic representation of the linoleic acid metabolism pathway, depicting the conversion of linoleate into various hydroperoxides, epoxides, and other derivatives. Colored highlights indicate changes in the pathway.

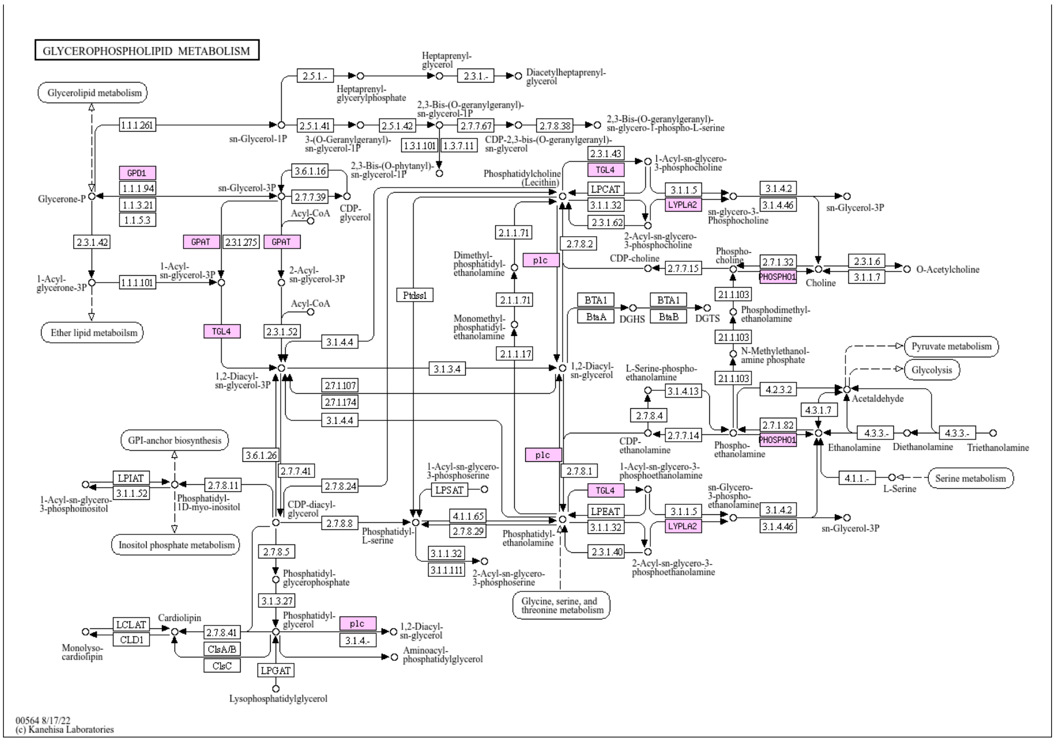

Figure S6. Schematic representation of the glycerophospholipid metabolism pathway, illustrating the conversion of glycerol-3-phosphate and related intermediates into various phospholipids and lysophospholipids. Colored highlights indicate changes in the pathway.

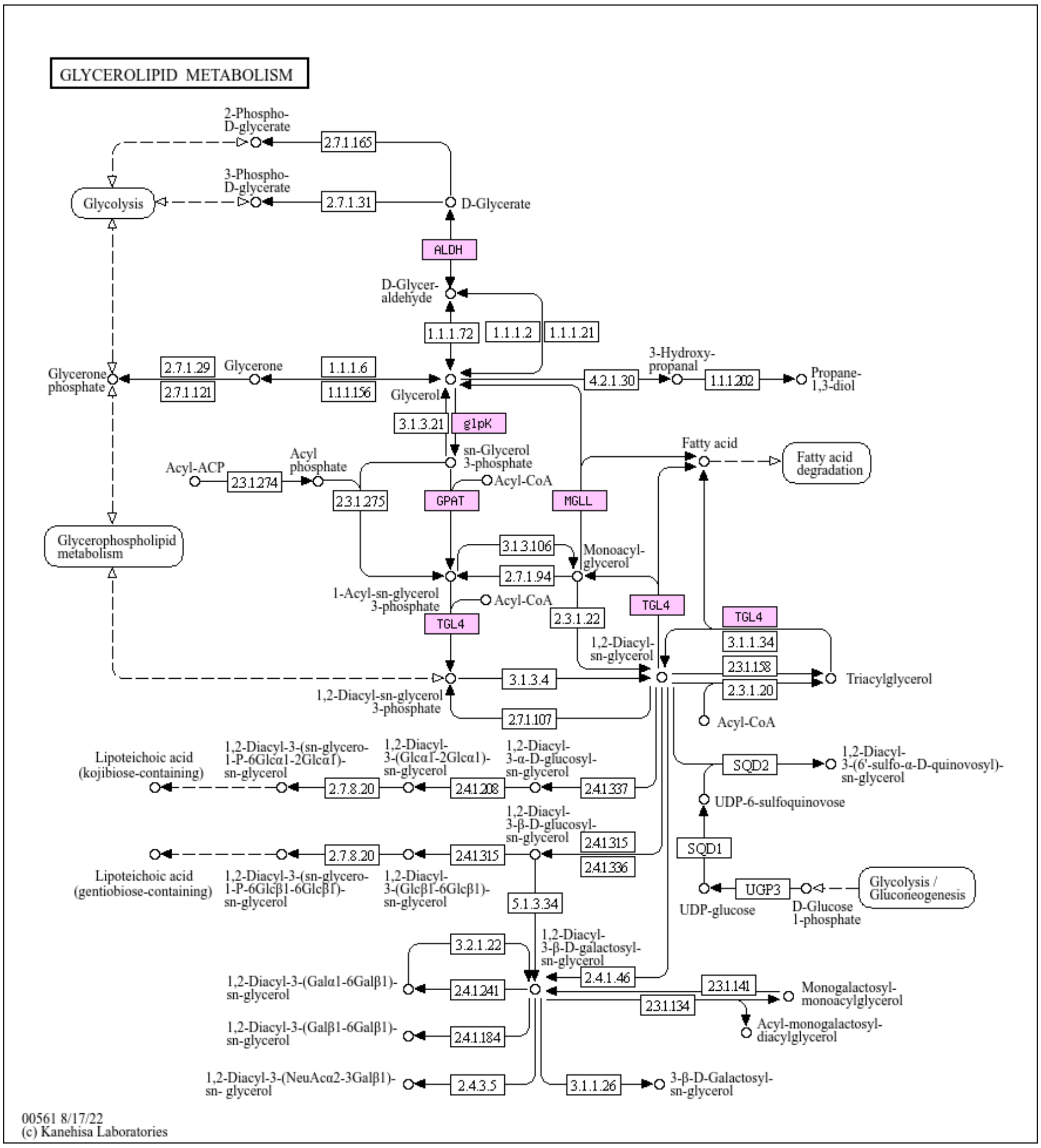

Figure S7. Schematic representation of the glycerolipid metabolism pathway, illustrating the conversion of glycerol-3-phosphate and related intermediates into various glycerolipids, including monoacylglycerol, diacylglycerol, and triacylglycerol. Colored highlights indicate changes in the pathway.

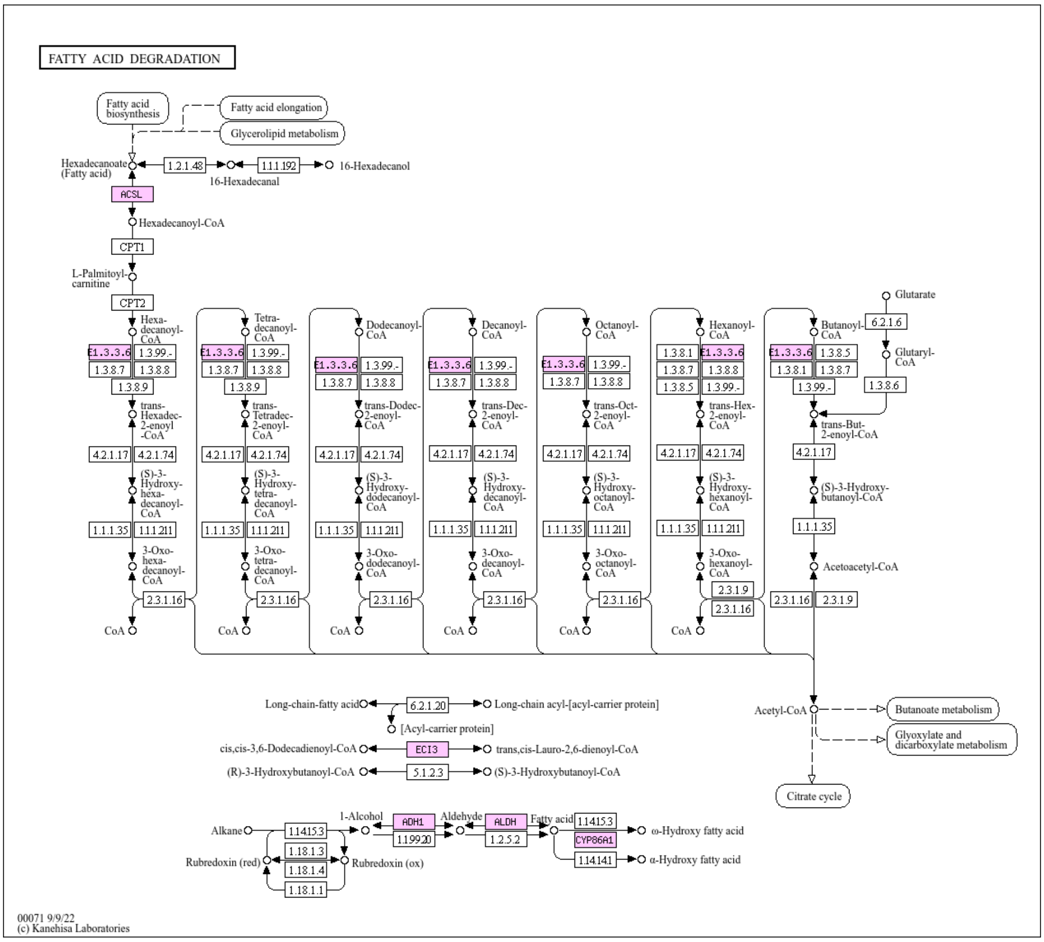

Figure S8. Schematic representation of the fatty acid degradation pathway, illustrating the breakdown of hexadecanoyl-CoA into acetyl-CoA and other short-chain acyl-CoAs through β-oxidation. Colored highlights indicate changes in the pathway.

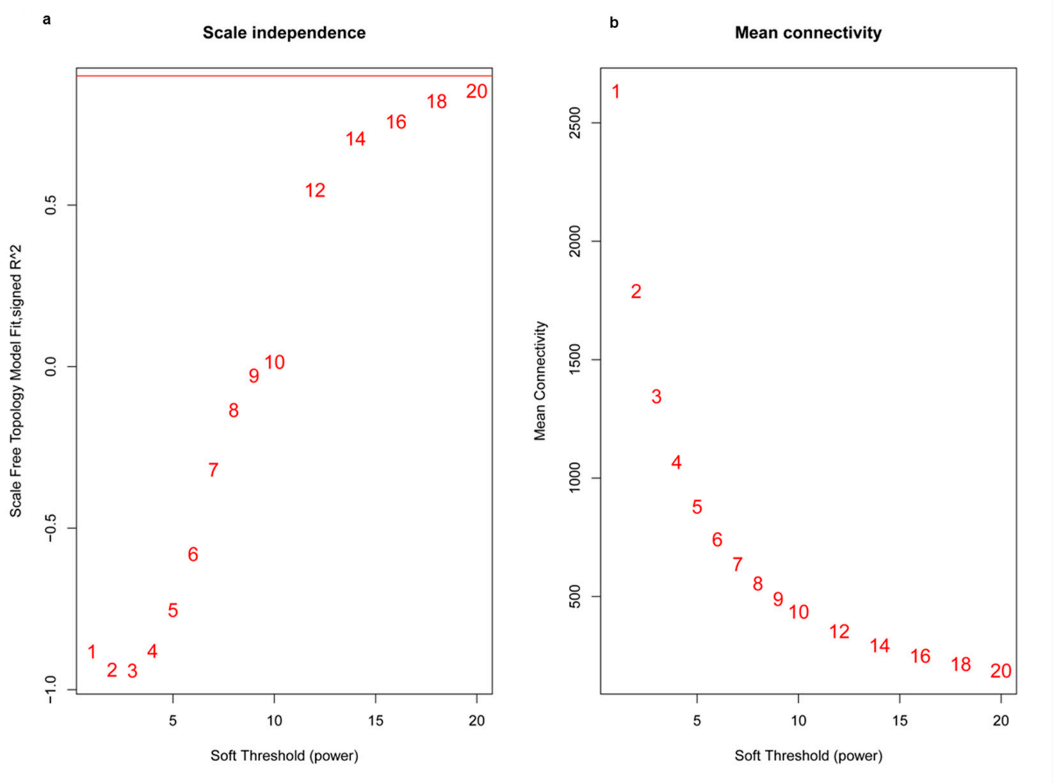

Figure S9. Analysis of scale independence and mean connectivity for network construction. (a) The left panel (Scale Independence) plots the scale-free topology model fit R^2 against soft-threshold power, with a threshold of 18 selected to achieve $R^2 \approx 0.9$, indicating a scale-free network. (b) The right panel (Mean Connectivity) shows mean connectivity decreasing with increasing soft-threshold power, illustrating the balance between network sparsity and connectivity at the selected threshold.

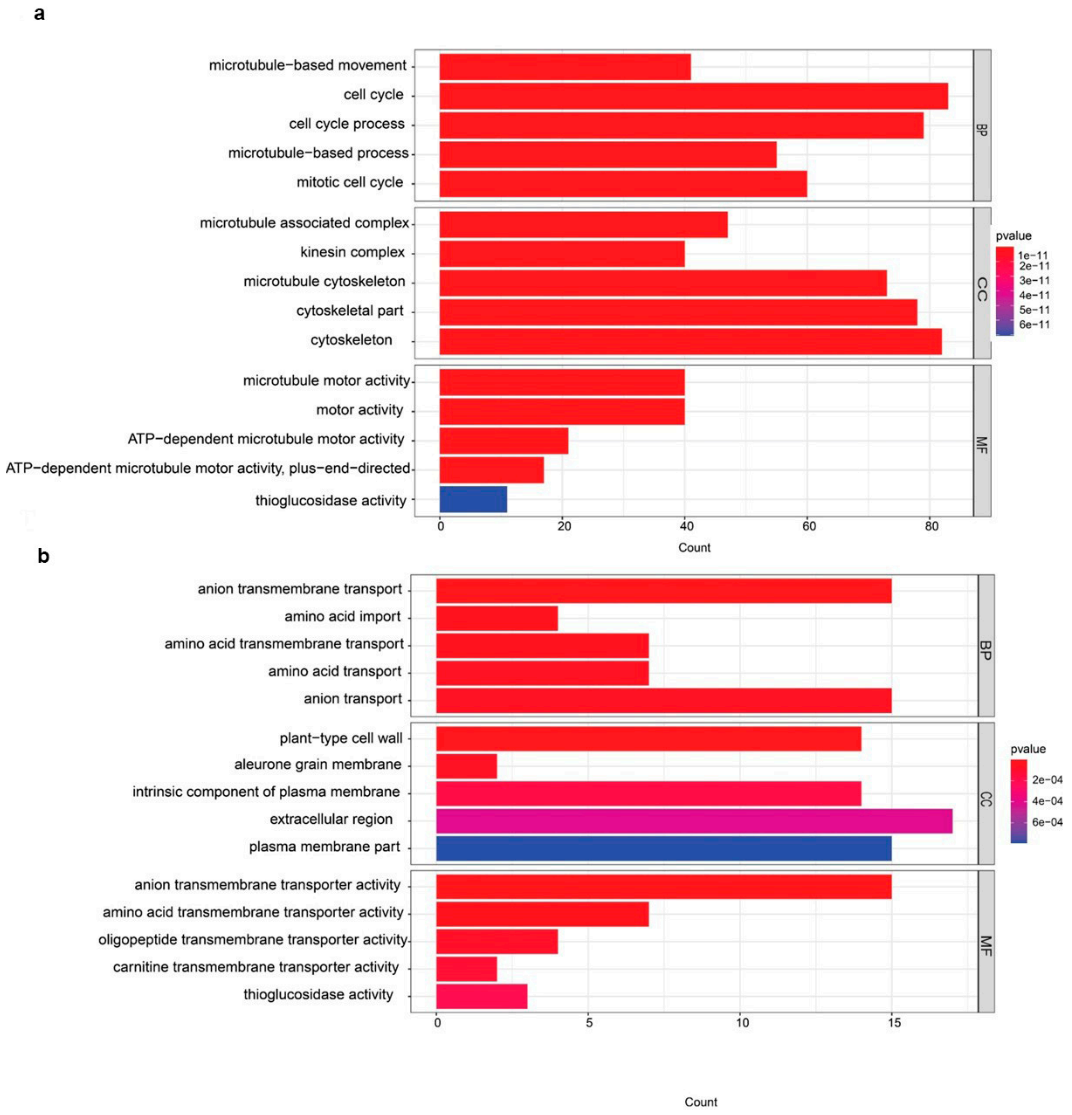

Figure S10. Gene Ontology (GO) enrichment analysis of genes in WGCNA modules. (a) GO enrichment of genes in the turquoise module. (b) GO enrichment of genes in the yellow module.

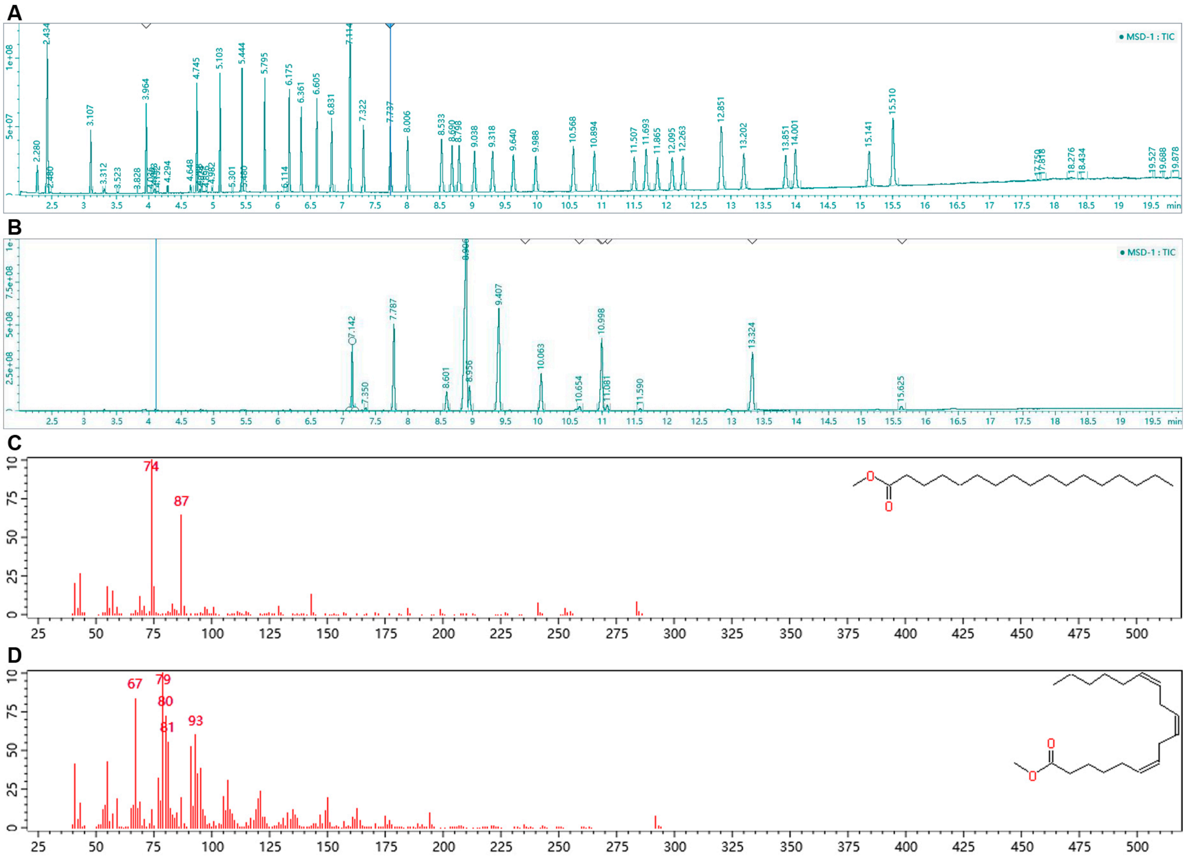

Figure S11. GC-MS total ion chromatograms (TICs) and mass spectral identification of commercial fatty-acid methyl ester standards and wild-type rapeseed. (A) GC-MS TIC of commercial fatty-acid methyl ester standards (GLC NESTLE 37 MIX, Acmec Biological), including C4:0, C6:0, C8:0, C10:0, C11:0, C12:0, C13:0, C14:0, C14:1, C15:0, C15:1, C16:0, C16:1, C17:0, C17:1, C18:0, C18:1, C18:1T, C18:2, C18:2T, C18:3, C18:3(γ), C20:0, C20:1, C21:0, C20:2, C22:0, C22:1, C20:3 (11,14,17-eicosatrienoate), C20:3 (homo-γ-linolenate), C20:4, C23:0, C22:2, C24:0, C20:5, C24:1, and C22:6. (B) GC-MS TIC of fatty-acid methyl esters derived from wild-type seeds. (C,D) Mass spectra used for the identification of C17:0 (C) and C18:3 (D) fatty-acid methyl esters.
